# Sleep differentially modulates synaptic vesicle protein 2A and spine density across cortical regions and development

**DOI:** 10.1016/j.isci.2026.116676

**Published:** 2026-07-15

**Authors:** Jing Ma, Alexandra Braun, Angela Oskamp, Lena Kricsfalussy-Hrabár, Nadja Hermes, Salma Djakkani, Ulrike Holz, Sabine Jakobs, Sabina Klein, Stefan Stüsgen, Philipp Krapf, Bernd Neumaier, Alexander Drzezga, Simone Beer, Andreas Bauer, Astrid Rollenhagen, Björn Kampa, David Elmenhorst

**Affiliations:** 1 Institute of Neurosciences and Medicine, Molecular Organization of the Brain (INM-2), Forschungszentrum Jülich, Jülich, Germany; 2 Institute of Neuroscience and Medicine, Nuclear Chemistry (INM-5), Forschungszentrum Jülich, Jülich, Germany; 3 Department of Nuclear Medicine, Faculty of Medicine and University Hospital Cologne, University of Cologne, Cologne, Germany; 4 Systems Neurophysiology, Institute of Zoology, RWTH Aachen University, Aachen, Germany; 5 JARA BRAIN, Institute of Neuroscience and Medicine (INM-10), Forschungszentrum Jülich, Jülich, Germany; 6 German Center for Neurodegenerative Diseases (DZNE), Bonn-Cologne, Germany; 7 Department of Neurology, Medical Faculty, Heinrich-Heine-University Düsseldorf, 40225 Düsseldorf, Germany

**Keywords:** synaptic plasticity, SV2A, dendritic spines, sleep, sleep deprivation, PET imaging

## Abstract

Sleep is proposed to regulate synaptic strength; yet, the underlying mechanisms remain debated under the synaptic homeostasis hypothesis (SHY). We examined synaptic vesicle glycoprotein 2A (SV2A) and dendritic spine density in mouse brain across conditions of physiological sleep and sleep deprivation. Spine density decreased during sleep and increased following sleep deprivation, consistent with the SHY. In contrast, [^18^F]SynVesT-1 positron emission tomography (PET) imaging and [^3^H]UCB-J autoradiography revealed reduced SV2A following sleep deprivation. Layer-specific analyses in somatosensory and visual cortices further supported coordinated regulation between SV2A and dendritic spines. Developmentally, adolescent mice exhibited higher baseline SV2A and spine density than adults, with greater SV2A sensitivity to sleep deprivation, whereas adults were more responsive to physiological sleep. Together, these findings reveal bidirectional regulation of pre- and postsynaptic compartments and highlight developmental differences in sleep-dependent synaptic organization.

## Highlights

• Physiological sleep and deprivation oppositely regulate spine density and SV2A
• SV2A and dendritic spines show coordinated and layer-specific modulation
• Adolescent mice exhibit higher baseline SV2A levels and spine density than adults
• Age differences shape sensitivity to sleep and sleep deprivation effects

## Graphical abstract

**Figure:**
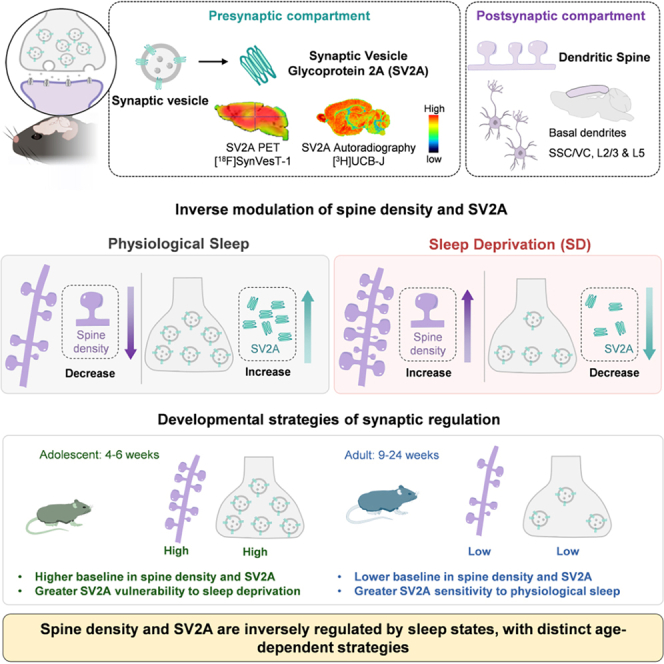

## Teaser

Molecular biology; Neuroscience; Cell biology

## Introduction

Sleep critically regulates synaptic plasticity. The synaptic homeostasis hypothesis (SHY) proposed that sleep renormalizes neural plasticity, counteracting the progressive strengthening of synaptic connections that occur during wakefulness.^1^^,^^2^ Substantial experimental evidence supports this framework. At the molecular level, sleep is associated with reduced expression of the GluR1 subunit of AMPA receptors; decreased phosphorylation of AMPAR, CaMKII, and GSK3β; and increased expression of synaptic inhibitory regulators, including CaMKIINα and calmodulin-dependent phosphatase.^3^^,^^4^^,^^5^^,^^6^ Electrophysiological studies have further demonstrated a reduced slope and amplitude of the cortical evoked responses during sleep.^7^^,^^8^^,^^9^ Structural studies using high-resolution microscopy have also shown reduction in the spine density and axon-spine interface.^10^^,^^11^^,^^12^^,^^13^^,^^14^ Importantly, this synaptic pruning during sleep appears selective, as learning-related spines are preferentially preserved.^15^^,^^16^ Despite the extensive use of synaptic markers in sleep research, each approach has inherent limitations. For example, spine density alone may not fully reflect functional synaptic connectivity, as spine morphology and size also influence synaptic efficacy, and not all spines form functional synapses. *In vitro* approaches may fail to capture dynamic circuit-level changes, whereas *in vivo* two-photon imaging is limited by sampling constraints. Together, methodological challenges highlight the complexity of characterizing sleep-dependent synaptic remodeling.

Furthermore, accumulating evidence suggests that sleep-dependent synaptic regulation is more complex than originally described by the SHY. Contrary to the simplified view that wakefulness promotes synaptic strengthening, sleep deprivation has been shown in some contexts to induce synaptic downregulation. For example, sleep deprivation can cause spine loss in hippocampal CA1 neurons, whereas rapid eye movement sleep stabilized motor cortex spines.^15^^,^^17^ At the molecular level, sleep deprivation enhances mTORC1-4EBP2-mediated protein synthesis while suppressing cAMP/PKA signaling.^18^^,^^19^ Electrophysiological studies further demonstrate that sleep deprivation impairs LTP-like plasticity in humans and inhibits LTP in rodents.^20^^,^^21^^,^^22^ In rodents, sleep-related synaptic alterations are reflected in synaptic efficacy without detectable changes in spine number.^23^ These divergent findings may partly arise from methodological differences, including the brain regions examined and the synaptic markers used. Supporting this view, a recent study has revealed substantial regional heterogeneity in spine remodeling across mouse amygdala subregions, highlighting that sleep-dependent synaptic strength regulation is not uniform across the brain.^24^

Moreover, most sleep research has focused primarily on postsynaptic mechanisms, whereas evidence for presynaptic regulation remains relatively limited, with only a few proteomic studies providing initial insights. For example, levels of the presynaptic protein synapsin I and its phosphorylated form increase following rapid eye movement sleep deprivation in rats.^25^ In contrast, other studies have reported that sleep deprivation markedly reduces several proteins involved in presynaptic endocytic processes.^26^^,^^27^ Given that presynaptic and postsynaptic components can be regulated independently with respect to timing, mechanisms, and function,^28^^,^^29^^,^^30^ resolving ongoing controversies surrounding sleep-mediated synaptic regulation will likely require an integrated understanding of both presynaptic and postsynaptic compartments.

Synaptic vesicle glycoprotein 2A (SV2A) is a major synaptic vesicle protein broadly expressed in both excitatory and inhibitory neurons. It plays an important role in neurotransmitter release, potentially through interacting with calcium-sensing machinery and vesicle-associated proteins such as synaptophysin-1, which regulate calcium-dependent exocytosis.^31^ Altered SV2A expression disrupts presynaptic plasticity. SV2A deletion reduces calcium-evoked exocytotic bursts and action potential-dependent GABAergic neurotransmission, linking SV2A deficiency to changes in the readily releasable pool (RRP).^32^^,^^33^ Indeed, several studies have reported reduced RRP size in SV2-deficient synapses, although others observed impaired evoked synaptic responses without detectable changes in RRP size.^31^^,^^34^ Conversely, overexpression of SV2A-EGFP has also been shown to decrease release probability.^35^ Together, these findings suggest that both reduced and elevated SV2A levels can disrupt synaptic transmission. Nevertheless, the mechanisms through which SV2A contributes to presynaptic plasticity and sleep-dependent synaptic modulation remain unclear. Beyond its physiological role, SV2A is the primary binding target of the antiepileptic drugs levetiracetam and brivaracetam. SV2A expression changes in several neuropsychiatric disorders, including epilepsy, Alzheimer’s disease, and depression, although whether these changes are causal or compensatory remains unresolved.^36^^,^^37^^,^^38^ Due to its widespread presynaptic localization and essential role in neurotransmission, SV2A has increasingly been used as a marker of synaptic density. The development of SV2A-specific positron emission tomography (PET) radiotracers has enabled *in vivo* quantification of synaptic density and revealed altered SV2A levels across multiple neurological and neurodegenerative disorders.^38^^,^^39^ Importantly, this approach enables non-invasive longitudinal assessment of synaptic changes *in vivo* and holds substantial translational potential. To date, PET imaging has been used to investigate the effects of sleep or sleep deprivation on adenosine receptors,^40^^,^^41^ noradrenaline receptors,^42^ serotonin 2A receptors,^43^ and β-amyloid accumulation,^44^ among others. However, evidence from SV2A PET studies remains relatively limited.

Given the controversy surrounding sleep-dependent synaptic changes, this study aimed to characterize dynamic alterations in presynaptic SV2A levels and postsynaptic dendritic spine density across the sleep-wake cycle and following sleep deprivation. The transition from adolescence to adulthood represents a critical period of brain structural and functional maturation and is closely linked to the vulnerability of neurodevelopmental and psychiatric disorders. Notably, previous studies have shown age-dependent differences in vulnerability to sleep deprivation,^45^ as well as in sleep-related dendritic spine turnover during sleep.^14^ Therefore, adolescent and adult mice were included for comparative analyses.

To this end, we combined *ex vivo* [^3^H]UCB-J SV2A autoradiography with Golgi impregnation of dendritic spines in the mouse brain, focusing on the basal dendrites of layer 2/3 (L2/3) and layer 5 (L5) pyramidal neurons in the primary somatosensory (SSC) and visual cortices (VC), key regions that receive extensive sensory inputs. These approaches were further complemented by longitudinal *in vivo* SV2A PET imaging using the radiotracer [^18^F]SynVesT-1. Additionally, we explored potential age-dependent regulatory differences in both SV2A expression and dendritic spine remodeling.

## Results

Using three circadian-aligned groups, synaptic regulation was examined via pairwise comparisons. Comparisons between light onset (ZT0; zeitgeber time 0) and light offset (ZT12; zeitgeber time 12) captured changes across the light phase. Comparisons between sleep deprivation (SD) group sampled at the light offset (ZT12) and the corresponding non-SD controls at the same circadian time isolated the effects of acute sleep loss independent of circadian influences.

### Spine density decreased during sleep and increased by SD

**Figure 1:**
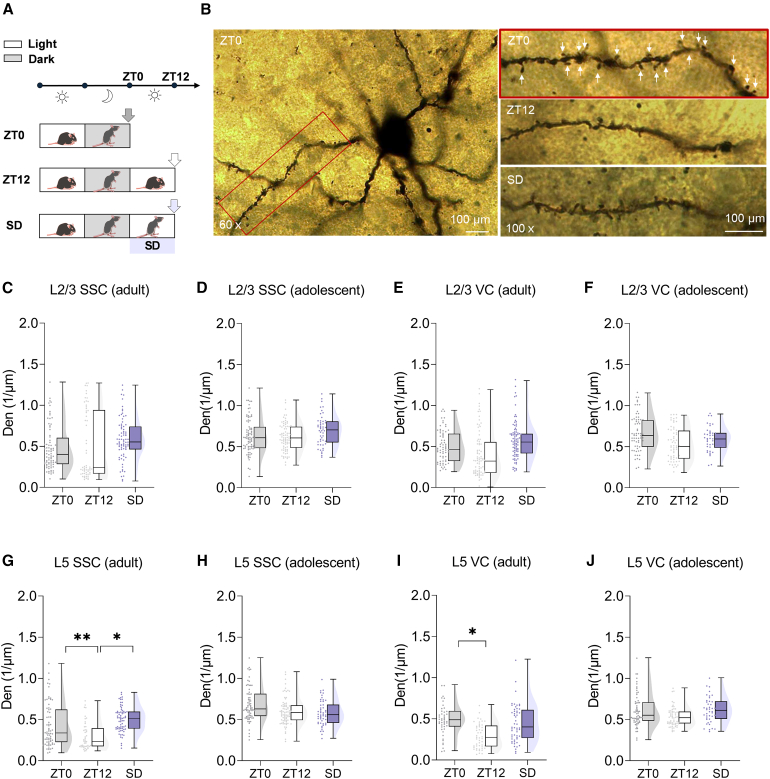
Dendritic spine analysis in pyramidal neurons (A) Schematic of the three experimental groups with tissue collection at ZT0 (gray), ZT12 (white), and ZT12 following sleep deprivation (SD, purple) by gentle handling. (B) Representative pyramidal neuron in L2/3 of the visual cortex (60×) and basal dendrite segments (100×) from ZT0, ZT12, and SD groups. Scale bars, 100 μm. Arrows indicate example spines on a ZT0 dendrite. (C–F) Spine density (spine number per μm, 1/μm) measured from reconstructed basal dendrites of pyramidal neurons in the L2/3 of SSC and VC of adult (9–24 weeks) and adolescent (4–6 weeks) mice. (G–J) Spine density (spine number per μm, 1/μm) measured from reconstructed basal dendrites of pyramidal neurons in the L5 of SSC and VC of adult (9–24 weeks) and adolescent (4–6 weeks) mice. Each data point represents the spine density of a single dendrite. Statistical significance was assessed using linear mixed-effects models (REML), with Sidak-adjusted post hoc comparisons. Degrees of freedom were estimated using the Kenward-Roger method, and Satterthwaite’s approximation was used for *t* tests. Significance levels are indicated as follows: ∗*p* < 0.05, ∗∗*p* < 0.01, ∗∗∗*p* < 0.001, and ∗∗∗∗*p* < 0.0001. Data are represented as mean ± SEM.

We analyzed basal dendritic spine density of pyramidal neurons from layers 2/3 (L2/3) and 5 (L5) of the SSC and VC in adolescent and adult mice from three groups (ZT0, ZT12, and SD), as shown in Figure 1A. All visible protrusions were counted under the light microscope, regardless of size or morphology (Figure 1B).

In general, spine density was lower in group ZT12 than ZT0 (see Table S3 in the supplemental information for detailed values). In the SSC, ZT12 vs. ZT0 comparisons showed decreases, except for adult L2/3 (Figure 1C), where spine density showed a slight, non-significant increase (+6.9%; ZT0: 0.48 ± 0.03 vs. ZT12: 0.51 ± 0.05; *p* = 0.4239). Notably, a significant reduction during the light phase was observed in adult L5 (Figure 1G; ZT12: 0.30 ± 0.02 vs. ZT0: 0.43 ± 0.03; −30.2%; *p* = 0.0069). In VC, a comparable, though non-significant, light-phase-associated decrease in spine density was observed, with both adult (Figure 1E) and adolescent mice (Figure 1F) showing similar reductions in L2/3 (adult: ZT12: 0.40 ± 0.03 vs. ZT0: 0.50 ± 0.02; −19.8%; *p* = 0.8359; adolescent: ZT12: 0.52 ± 0.03 vs. ZT0: 0.67 ± 0.03; −21.3%; *p* = 0.2894). Notably, in VC, the effects of light across age and cortical layers were similar to those observed in SSC, with a significant decrease only observed in adult L5 (Figure 1I; ZT12: 0.30 ± 0.02 vs. ZT0: 0.49 ± 0.03; −40.0%; *p* = 0.0397), whereas no significant change was observed in the young group (Figure 1J).

In contrast to changes across the light phase, sleep deprivation induced a general increase in spine density across all regions and layers, although not all changes reached statistical significance. A statistically significant difference was observed only in SSC L5, with a 68.5% increase (Figure 1G; SD: 0.50 ± 0.02 vs. ZT12: 0.30 ± 0.02; *p* = 0.0113).

In addition, we compared SD animals with those sampled at the ZT0 to assess the overall impact of sleep deprivation on circadian-regulated structural changes. No significant differences were observed between the ZT0 and SD in either adult or young mice (*p* > 0.05).

### SV2A increased during sleep and decreased by SD

**Figure 2:**
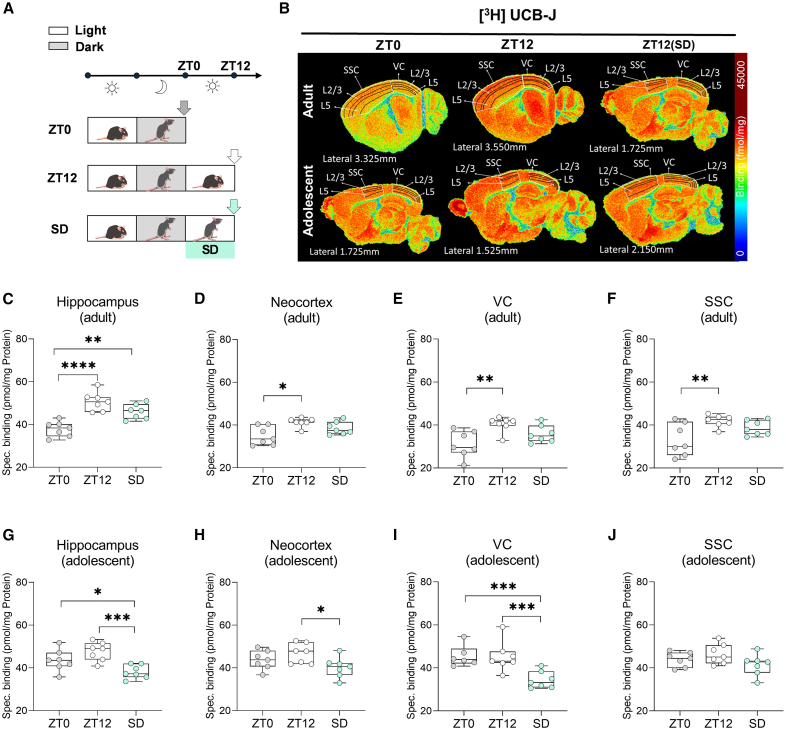
Regional SV2A expression quantified by [^3^H]UCB-J autoradiography (A) Schema of the three experimental groups with tissue collection at ZT0 (gray), ZT12 (white), and ZT12 after sleep deprivation (SD, green) by gentle handling. (B) Representative autoradiograms with brain region localization referenced to the Allen Mouse Brain Atlas, including neocortex, hippocampus, and L2/3 and L5 of SSC and VC. (C–J) Quantification of [^3^H]UCB-J specific binding (fmol/mg protein) in (C–F) adolescent (4–6 weeks) and (G–J) adult (9–24 weeks) mice. Statistical significance was assessed using linear mixed-effects models (REML), with Sidak-adjusted post hoc comparisons. Degrees of freedom were estimated using the Kenward-Roger method, and Satterthwaite’s approximation was used for *t* tests. Significance levels are indicated as follows: ∗*p* < 0.05, ∗∗*p* < 0.01, ∗∗∗*p* < 0.001, and ∗∗∗∗*p* < 0.0001. Data are represented as mean ± SEM.

In addition to spine density, we also quantified the presynaptic SV2A via autoradiography in the hemispheres contralateral to the Golgi-stained sections. It provided data from additional brain regions; the hippocampus was included in the analysis alongside the SSC and VC (Figures 2A and 2B). Overall, SV2A showed a trend opposite to that of spine density across the light phase and following sleep deprivation.

In adult mice, hippocampal [^3^H]UCB-J specific binding is significantly higher at ZT12 than ZT0 (ZT12: 50.7 ± 1.7 vs. ZT0: 37.9 ± 1.3 pmol/mg; *p* < 0.0001; +34.0%; Figure 2C). Similarly, the binding in the neocortex of adult groups differed by 18.3% between ZT12 and ZT0 across the light phase (ZT12: 41.3 ± 0.8 vs. ZT0: 34.9 ± 1.6 pmol/mg; *p* = 0.0135; Figure 2D). Within the neocortex of adult, significant higher [^3^H]UCB-J binding was observed at the end of the light phase than at its onset in both the VC (ZT12: 40.4 ± 1.4 vs. ZT0: 31.1 ± 2.3 pmol/mg; *p* = 0.0026; +30.0%; Figure 2E) and SSC (ZT12: 41.9 ± 1.0 vs. ZT0: 33.1 ± 2.8 pmol/mg; *p* = 0.0045; +26.8%; Figure 2F). Similarly, adolescent mice showed a sleep-related trend but no significant enhancement (ZT12 vs. ZT0; all *p* > 0.05; Figures 2G–2J).

Following sleep deprivation, [^3^H]UCB-J binding in young mice was significantly reduced compared with the normal-sleep (ZT12) in both hippocampus (SD: 38.2 ± 1.2 vs. ZT12: 47.7 ± 1.6 pmol/mg; *p* = 0.0001; −20.0%; Figure 2G) and neocortex (SD: 40.2 ± 1.8 vs. ZT12: 46.6 ± 1.8 pmol/mg; *p* = 0.0129; −13.8%; Figure 2H). Within neocortical subregions, only the VC showed a significant effect (SD: 34.4 ± 1.5 vs. ZT12: 45.2 ± 2.6 pmol/mg; *p* = 0.0004; −24.0%; Figure 2I). In adult mice, all regions showed a similar but non-significant downward trend, with reduced in binding following sleep deprivation (SD vs. ZT12; Figures 2C–2F).

Interestingly, the [^3^H]UCB-J binding measured at the light onset (ZT0) and after prolonged wakefulness (SD) revealed clear age-dependent differences. In young mice, binding was consistently lower in the SD group compared to the ZT0 group, with a significant reduction observed in the hippocampus (*p* = 0.0490; Figure 2G). Although no significant changes were observed in the neocortex overall (*p* = 0.3022; Figure 2H), SD led to a marked decrease in the VC (*p* = 0.0002; Figure 2I) compared to ZT0, whereas no difference was detected in the SSC (*p* = 0.7135; Figure 2J). In adults, the pattern reversed, with generally higher binding following SD than ZT0. The hippocampus showed a significant higher binding in SD (*p* = 0.0012; Figure 2C), while the neocortex showed a similar tendency but not significant (*p* = 0.2601; Figure 2D), and similar trends were also found in VC and SSC (Figures 2E and 2F). Together, we observed an increase in the presynaptic SV2A density across the light phase and that this effect is attenuated or reversed by prolonged wakefulness.

To correspond with the spine samples collected from L2/3 and L5, we also delineated regions of interest (ROIs) in SSC and VC L2/3 and L5 for analysis (Figures S2 and S3), with detailed values reported in Table S4. Consistent with the analysis in the entire SSC region (Figures 2F and 2J), light-phase-associated significant increases in [^3^H]UCB-J binding were observed in L2/3 and L5 of adults (Figures S2B and S2D). L2/3 increased by 22.8% (ZT12: 42.5 ± 1.3 vs. ZT0: 34.6 ± 2.4 pmol/mg; *p* = 0.0267) and L5 by 35.3% (ZT12: 45.8 ± 1.1 vs. ZT0: 33.9 ± 2.7 pmol/mg; *p* = 0.0004).

In adult VC, where light-phase increases were observed (Figure 2E), significant changes were limited to L5 (+31.4%, ZT12: 42.5 ± 1.2 vs. ZT0: 32.4 ± 2.6 pmol/mg; *p* = 0.0029, Figure S3D), whereas L2/3 showed a non-significant increase (+20.1%, ZT12: 37.8 ± 1.8 vs. ZT0: 31.5 ± 2.2 pmol/mg; *p* = 0.0987, Figure S3B).

Layer-specific analysis of sleep deprivation effects showed a similar age-specific pattern. In the VC, the SD-induced decrease in [^3^H]UCB-J binding was significant only in young mice, with L2/3 declining by 26.1% (*p* = 0.0004) and L5 by 24.6% (*p* = 0.0002), as shown in Figures S3A and S3C. In adult VC, no significant decreases in [^3^H]UCB-J binding were observed in either layer following sleep deprivation.

### [^18^F]SynVesT-1 PET: SV2A deceased after sleep deprivation

**Figure 3:**
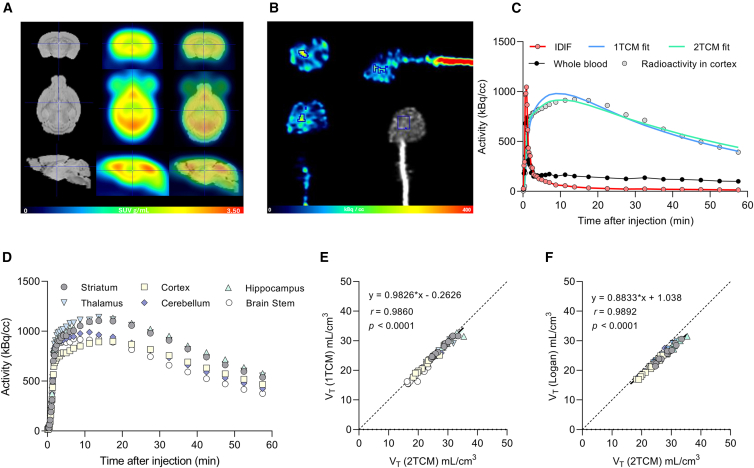
[^18^F]SynVesT-1 PET imaging and kinetic analysis in mice (*n* = 14, 9–16 weeks) (A) Representative SUV images (30–60 min) with Mirrione-T2 atlas overlay in coronal (top), horizontal (middle), and sagittal (bottom) planes. (B) Early-phase PET images and corresponding IDIF (blue curve) derived from cardiac VOI (>50% max uptake within 10 mm^3^). Shown in coronal, sagittal, transverse planes and coronal maximum intensity projection (MIP) (lower right). (C) Kinetic modeling of cortical time-activity data using 1TCM (blue) and 2TCM (green). Whole-blood curve (black) scaled from IDIF (red); measured data shown as gray points. (D) Time-activity curves in striatum, cortex, hippocampus, thalamus, and cerebellum over 60 min; symbols denote regions. (E and F) Regression lines compare 2TCM with (E) 1TCM and (F) Logan estimates; dashed lines indicate identity (y = x).

To further evaluate the change in presynaptic SV2A density, we used longitudinal SV2A PET imaging with [^18^F]SynVesT-1 to evaluate the effects of acute sleep deprivation and map its spatial distribution across the mouse brain. Regional standardized uptake value (SUV, 30-60 min post injection) maps showed high uptake in the striatum, hippocampus, and thalamus, with relatively low uptake in the cortex and cerebellum, and minimal uptake in the brainstem (Figure 3A). Time-activity curves reached their peak at approximately 5 min and exhibited gradual washout after 20 min (Figure 3D).

Using an input function derived from metabolite-corrected cardiac images (image-derived input function [IDIF]; Figure 3B), we estimated the total volume of distribution (V_T_) via one-tissue-compartment (1TCM), two-tissue-compartment (2TCM), and Logan plot analyses (Figure 3C). V_T_ values from 1TCM and Logan plots correlated strongly with 2TCM estimates (Figures 3E and 3F), and all three methods yielded comparable coefficients of variation (%COV; Table S5), with 2TCM showing the lowest variability. Bland-Altman analysis, however, indicated that 1TCM and Logan plots systematically underestimated V_T_ relative to 2TCM (Figure S7), leading us to select 2TCM with IDIF for V_T_ estimation.

**Figure 4:**
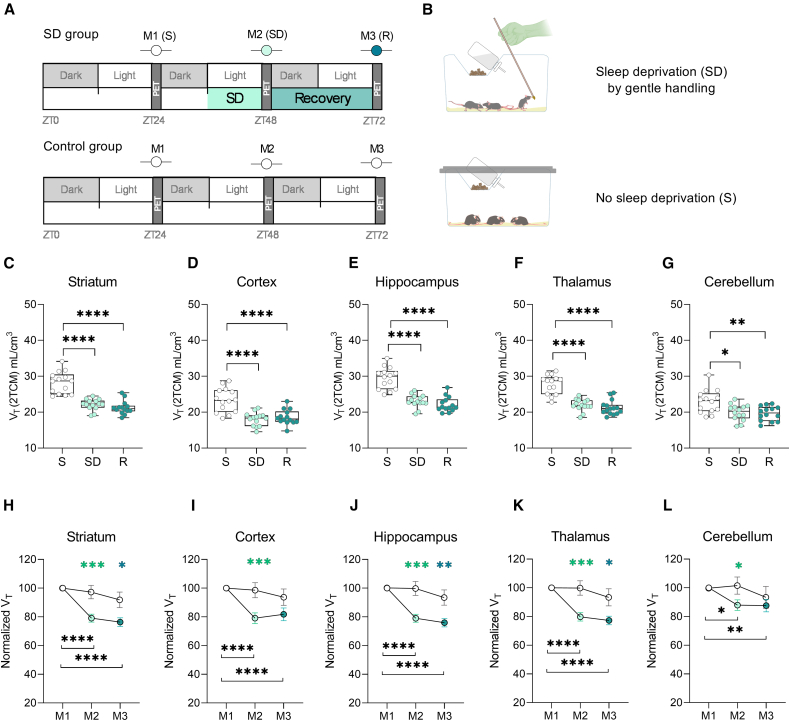
Effects of sleep deprivation on SV2A assessed by *in vivo* [^18^F]SynVesT-1 PET Experimental timelines for longitudinal PET imaging in the (A) SD group (9–16 weeks) and (B) control group (13–15 weeks). SD group underwent three scans: after sleep (S, M1), after sleep deprivation (SD, M2), and after 24 h recovery (R, M3). Controls were scanned at matched time points (M1, M2, and M3) following normal sleep. (C–G) Total distribution volume (V_T_, 2TCM) of [^18^F]SynVesT-1 in striatum, cortex, hippocampus, thalamus, and cerebellum for SD mice across AS (white), SD (light green), and R (dark green). (H–L) Normalized V_T_ of SD and control groups. Values were normalized to the first measurement (S or M1 = 100%). Statistical significance was assessed using linear mixed-effects models (REML), with Sidak-adjusted post hoc comparisons. Degrees of freedom were estimated using the Kenward-Roger method, and Satterthwaite’s approximation was used for *t* tests. Significance levels are indicated as follows: ∗*p* < 0.05, ∗∗*p* < 0.01, ∗∗∗*p* < 0.001, and ∗∗∗∗*p* < 0.0001. Data are represented as mean ± SEM.

Longitudinal [^18^F]SynVesT-1 PET scanning in adult mice over 3 consecutive days revealed lower SV2A availability following sleep deprivation (SD) (Figure 4A). Quantitatively, V_T_ showed significant reductions following SD across all examined regions: striatum (SD: 22.1 ± 0.4 vs. AS: 28.2 ± 0.8, *p* < 0.0001, −21.6%), cortex (SD: 18.0 ± 0.5 vs. AS: 23.3 ± 0.9; *p* < 0.0001; −22.7%), hippocampus (SD: 23.1 ± 0.5 vs. AS: 29.5 ± 0.8; *p* < 0.0001; −21.8%), thalamus (SD: 22.0 ± 0.4 vs. AS: 27.8 ± 0.7; *p* < 0.0001; −20.8%), and cerebellum (SD: 19.9 ± 0.6 vs. AS: 22.8 ± 0.8; *p* = 0.0138; −13.1%) (Figures 4C–4G).

After a 24-h recovery period (R), V_T_ values approximated the SD group and exhibited mild changes. The V_T_ decreases of 3.9% in striatum (R: 21.3 ± 0.5 vs. SD: 22.1 ± 0.4; *p* = 0.7001), 4.1% in hippocampus (R: 22.1 ± 0.6 vs. SD: 23.1 ± 0.5, *p* = 0.6959), 3.3% in thalamus (R: 21.3 ± 0.6 vs. SD: 22.0 ± 0.4, *p* = 0.7959), and 1.5% in cerebellum (R: 19.7 ± 0.5 vs. SD: 19.9 ± 0.6, *p* = 0.9961), and a slight 3.1% increase in cortex (R: 18.6 ± 0.6 vs. SD: 18.0 ± 0.5, *p* = 0.9071).

To exclude potential procedural effects on synaptic density, a parallel group of animals (*n* = 7, male, 13–15 weeks) underwent three consecutive days of PET scanning with normal sleep (M1, M2, M3, Figure 4A). V_T_ values revealed a gradual decline in tracer accumulation across all regions, with no significant changes observed between M1 and M2 (Figure S8). On M3, availability of [^18^F]SynVesT-1 was lower than M1 (striatum: *p* = 0.0104; cortex: *p* = 0.0809; hippocampus: *p* = 0.0357; thalamus: *p* = 0.0323; cerebellum: *p* = 0.0751). For clarity, these post hoc multiple comparisons are described here and are not indicated directly in Figure 4.

Importantly, the magnitude of this decline was smaller than the reduction in the SD group following sleep deprivation. To account for the possible inherent variability across different animal batches, the V_T_ values were normalized to M1 or S (set as 100 percent). The SD group exhibited markedly greater reductions than controls in the second scan (SD vs. M2) across all regions: striatum (M2: 97.3 ± 4.7; SD: 79.3 ± 2.7; *p* = 0.0002; light green asterisks in Figure 4H), cortex (M2: 99.7 ± 4.9; SD: 78.9 ± 3.6; *p* = 0.0005; light green asterisks in Figure 4I), hippocampus (M2: 98.6 ± 5.3; SD: 79.0 ± 2.8; *p* = 0.0001; light green asterisks in Figure 4J), thalamus (M2: 99.9 ± 5.1; SD: 80.0 ± 2.8; *p* = 0.0001; light green asterisks in Figure 4K) and cerebellum (M2: 101.9 ± 6.0; SD: 88.5 ± 3.5; *p* = 0.0288; light green asterisks in Figure 4L). Within the SD group, normalized V_T_ values decreased significantly after SD compared with the initial baseline (AS) in all regions (striatum: *p* < 0.0001; cortex: *p* < 0.0001; hippocampus: *p* < 0.0001; thalamus: *p* < 0.0001; cerebellum: *p* = 0.0147; black asterisks in Figures 4H–4L). By the third scan, SD mice maintained consistently greater V_T_ reduction than controls in multiple brain regions (striatum: *p* = 0.0251; hippocampus: *p* = 0.0095; thalamus: *p* = 0.0180; dark green asterisks in Figures 4H–4L).

### Reciprocal regulation of presynaptic and postsynaptic compartments

**Figure 5:**
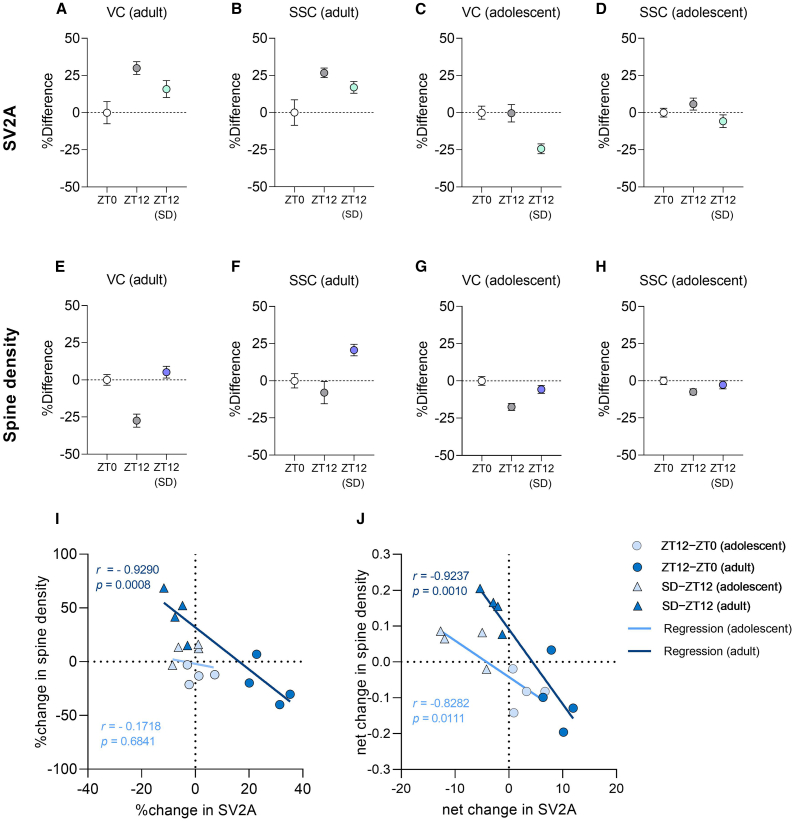
Bidirectional changes in presynaptic SV2A and postsynaptic spine density across circadian time and sleep deprivation Percentage difference of (A–D) presynaptic SV2A expression levels (quantified by [^3^H]UCB-J autoradiographic specific binding) and (E–H) postsynaptic spine density in the SSC and VC of young and adult mice at ZT0 (light onset), ZT12 (light offset), and ZT12 following sleep deprivation (SD), expressed relative to the mean value of the ZT0. Calculation was performed relative to the mean of the ZT0 group as shown in equation: %Difference_*(i)*_ = (value_*(i)*_ - mean_*(ZT0)*_)/mean_*(ZT0)*_ ∗ 100%. Linear regression between (I) percent change and (J) absolute values of SV2A levels and spine density obtained in layers 2/3 and 5 of the SSC and VC from young (light blue) and adult (dark blue), comparing ZT12 vs. ZT0 (dot) and SD vs. ZT12 (triangle) conditions. Data are represented as mean ± SEM.

Our findings revealed a consistent pattern of bidirectional regulation: presynaptic SV2A increased during the light-on phase while postsynaptic spine density decreased, whereas sleep deprivation had the opposite effect. To compare SV2A and spine regulation, values were normalized to those evaluated at ZT0. As shown in Figure 5, SV2A and spine density in SSC and VC exhibited state-dependent coordination across circadian time points and following prolonged wakefulness. In adult VC, sleep induced a 27.4% decrease in spine density with a 30.0% increase in SV2A (ZT0 vs. ZT12; Figures 5A and 5E), consistent across cortical layers: spine density decreased in L2/3 (−19.8%; Figure S9A) and L5 (−39.9%; Figure S9B), while SV2A increased in L2/3 (+20.1%; Figure S9E) and L5 (+31.4%; Figure S9F). Young mice displayed a similar bidirectional regulation but with smaller changes. In SSC, young mice showed a 7.4% decrease in spine density paired with a 5.7% increase in SV2A (Figures 5D and 5H). In L5 of young SSC (Figures S10D and S10H), a 12.0% spine reduction corresponded to a 7.2% SV2A rise. In L5 of young VC (Figures S9D and S9H), spine density decreased by 13.3% over the 12-h light period, accompanied by a 14.9% increase in SV2A.

Subsequently, we analyzed the correlation between proportional changes in spine density and SV2A during the physiological sleep phase [(ZT12-ZT0)/ZT0 × 100%] and following sleep deprivation [(SD- ZT12)/ZT12 × 100%] using data from L2/3 and L5 of SSC and VC (Tables S3 and S4). Percentage changes in spine density and SV2A showed a strong linear relationship particularly in adult mice (Pearson’s *r* = 0.9290, *p* = 0.0008, Figure 5I, dark blue), whereas young mice demonstrated a non-significant correlation (Pearson’s *r* = 0.1718, *p* = 0.6841, Figure 5I, light blue).

Given that reference values (ZT0) differed across age groups, normalization may obscure the true magnitude of synaptic changes. Therefore, we further compared the absolute net changes in tracer binding (pmol/mg protein) and spine density (spines/μm). Net changes showed a significant negative correlation between spine density and SV2A alterations both in young mice (Pearson’s *r* = −0.83, *p* = 0.0111, light blue) and in adults (Pearson’s *r* = −0.92, *p* = 0.0010, dark blue) as shown in Figure 5J.

### Age-specific differences in sleep-dependent synaptic regulation

#### Higher basal pre- and postsynaptic density in adolescent mice

In addition to group differences, the linear mixed model (LMM) analysis revealed a significant age-dependent effect in the quantification of presynaptic SV2A expression (F (1, 36) = 18.61, *p* = 0.0001) and significant age-dependent effects in spine density (F (1, 20.24) = 13.04, *p* = 0.0017). Post hoc comparisons were then conducted to examine age-related differences across groups, with a particular focus on sleep-wake transitions.

At ZT0, adolescent mice showed higher, though not statistically significant, dendritic spine densities than those seen in adults: by 46.7% in SSC (*p* = 0.1236, Figure S1A) and 28.0% in VC (*p* = 0.0927, Figure S1C). At the light offset (ZT12), spine density in young mice was 45.2% higher than adult in SSC (*p* = 0.0008, Figure S1B) and 47.2% higher in VC (*p* = 0.0217, Figure S1D). Autoradiography further confirmed higher SV2A expression in young mice (Figure S4). Specifically, significantly higher specific binding was observed in young mice than in adults across regions at ZT0 (neocortex: *p* = 0.0001, 25.2% higher; hippocampus: *p* = 0.0117, 14.8% higher; SSC: *p* = 0.0001, 32.8% higher; VC: *p* < 0.0001, 46.1% higher). At ZT12, young mice continued to show significantly higher SV2A expression in the neocortex (*p* = 0.0162, 13.0% higher), while trends toward higher expression were observed in the SSC (*p* = 0.0923; 10.8% higher) and VC (*p* = 0.0724; 11.9% higher), although these differences did not reach statistical significance.

#### Age-dependent sensitivity of SV2A

Although the LMM did not reveal a significant group × age interaction for spine density (F (2, 20.23) = 0.77, *p* = 0.4748), spine remodeling was more pronounced in adults compared to adolescents, with significant light- and SD-associated effects observed only in adult mice. Moreover, although these changes did not reach statistical significance, the percentage change in spine density was greater in adults than in adolescents (Table S3).

In addition to simple age-related differences in spine density and SV2A expression, the LMM analysis revealed a group × age interaction for SV2A measures (F (2, 36) = 5.21, *p* = 0.0102).

As shown in Figure 2, across the 12-h light phase, alterations in SV2A were significant only in adults, and the percentage changes in adults were generally greater than in young mice (Table S4). In adults, SV2A expression at ZT12 relative to ZT0 was 18.3% higher in the neocortex and 34.0% higher in the hippocampus. In contrast, young mice showed only modest differences relative to ZT0, with values 6.7% higher in the neocortex and 9.6% higher in the hippocampus. Similarly, in subcortical regions, adults showed values 26.8% higher in the SSC and 30.0% higher in the VC at ZT12 than ZT0, whereas young mice showed only modest differences, with 5.7% higher in the SSC and essentially unchanged at −0.4% in the VC (Table S4).

The effects of sleep deprivation occurred exclusively in young mice. Sleep deprivation exerted an inhibitory effect on SV2A expression, as indicated by lower levels in the SD condition relative to ZT12, with a more pronounced reduction in adolescents. In adolescent mice, SV2A levels were reduced relative to ZT12 by 13.8% in the neocortex, 20.0% in the hippocampus, 10.9% in the SSC, and 24.0% in the VC, whereas in adults the corresponding decreases were smaller (neocortex: 6.6%; hippocampus: 9.4%; SSC: 7.7%; VC: 11.3%).

Overall, these results demonstrated a clear age- and region-dependent divergence in the response to sleep deprivation: in young mice, sleep deprivation predominantly induced reductions in SV2A, with the greatest vulnerability in the hippocampus and VC, whereas in adults, sleep was associated with prominent SV2A increases. Together, these findings highlight differential sensitivity of SV2A to sleep-wake regulation across development.

#### Age-dependent regulation in prolonged wakefulness

Comparisons between SD and ZT0 groups represent the overall effects of prolonged sleep across the circadian light phase and reveal a marked age-dependent divergence. In both presynaptic (SV2A) and postsynaptic (spine density) measures, SD was associated with reductions in young mice but increases in adults. Although statistical significance was reached only in selected regions, this trend was consistent across both the SSC and VC (Figure 5). Notably, despite SD-induced increases in adults (vs. ZT0), SV2A levels and spine density did not exceed those of young mice at ZT0 and in some cases remained lower.

#### Layer-specific analysis

Significant layer-specific effects were detected for both spine density (F(1, 1487) = 19.57, *p* < 0.0001) and SV2A levels (F(1, 108) = 27.85, *p* < 0.0001), along with significant group × layer interactions for spine density (F(1, 1487) = 5.12, *p* = 0.0061) and SV2A levels (F(1, 108) = 7.21, *p* = 0.0011).

At baseline circadian time points (ZT0 and ZT12 without intervention), spine density was generally comparable between L2/3 and L5. In young mice, no significant differences in spine density were observed across cortical layers (Figures S5A and S5B). In adult mice, at ZT0, there was no significant difference in spine density between L2/3 and L5 neurons. However, following the light exposure phase, at ZT12, spine density in L5 neurons was significantly lower than in L2/3 neurons in the SSC (Figure S5C, *p* < 0.0001) and VC (Figure S5D, *p* = 0.0028). This difference is primarily attributable to spine downregulation during this phase, which was more pronounced in L5 than in L2/3 neurons. This trend is further supported by the percentage changes shown in Table S3 and by the quantitative analysis of spines across layers in Figure 1.

We also assessed the layer specificity of SV2A expression in parallel (Figure S6). At ZT0, no significant difference in SV2A expression was observed between L2/3 and L5 neurons. However, after 12 h of light exposure, SV2A expression was upregulated in both layers, with a greater increase in L5 than in L2/3, resulting in higher SV2A levels in L5. Consistent with this, circadian analyses across cortical layers showed that sleep-related SV2A increases were primarily observed in L5 rather than L2/3 (Figures S2 and S3).

In this study, we did not directly compare SV2A expression or spine density between L2/3 and L5 in the sleep deprivation (SD) group. Instead, we analyzed synaptic regulation within each layer across conditions (ZT0, ZT12, and SD), which provides a clearer view of SD-related regulatory patterns. As shown in Figure 1, regulations in spine density in adult mice were more pronounced in L5 than in L2/3, suggesting that L5 spines may be more susceptible to sleep- or SD-related modulation. Similarly, sleep-related SV2A enhancement during the light phase were mainly observed in L5 (Figures S2D and S3D). However, SD-related changes in SV2A expression did not exhibit clear layer specificity.

Overall, the regulatory SV2A trends related to light and sleep deprivation in L2/3 and L5 were consistent with observations at the whole-brain level. The age-dependent regulation of SV2A in both layers also aligned with the pattern described above. Sleep-related SV2A enhancement was most significant in adult mice, while the increase in SV2A expression following sleep deprivation was most significant in young mice. Overall, these findings underscore both the heightened responsiveness of adult spine and SV2A during the light phase and the critical involvement of L5.

## Discussion

### Sleep differentially affects SV2A and spine density

Our findings reveal a bidirectional regulatory strategy operating across pre- and post-synaptic compartments over the sleep-wake cycle. In this study, measurements taken at the light onset and offset in mice, corresponding to the physiological sleep period, showed a reduction in spine density alongside an increase in [^3^H]UCB-J binding, indicating net spine pruning and an overall upregulation of SV2A expression during spontaneous sleep. In contrast, the sleep deprivation group exhibited changes in the opposite direction, with increased spine density and decreased SV2A levels, further highlighting the regulatory effects of sleep observed during the light phase.

Additionally, our *in vivo* longitudinal PET imaging consistently indicated that SV2A levels were downregulated after sleep deprivation, which agrees with the earlier reports that sleep deprivation disrupts presynaptic function. For instance, 6 h of sleep deprivation in mice resulted in decreased synaptosomal-associated protein 25 (SNAP25) levels in the cerebral cortex and basal forebrain, as well as in the abundance of synaptophysin 1b and synaptophysin.^46^ In parallel, syntaphilin, which inhibits SNARE complex assembly and blocks synaptic vesicle fusion, increases following sleep deprivation.^27^ These coordinated declines in protein expression may be driven by altered protein synthesis and transport during sleep loss and altered SV2A levels are thought to be correlated with potential changes in synaptic vesicle numbers or vesicle recycling efficiency within the presynaptic compartment.^47^^,^^48^^,^^49^ Vesicle-cycling proteins have been shown to exhibit circadian rhythmicity. For example, the vesicle-trafficking N-ethylmaleimide-sensitive fusion protein (NSF) and synaptic protein II both increase during spontaneous sleep within the light phase,^50^ consistent with the sleep-related elevation of SV2A observed in this study. Taken together, these circadian fluctuations in SV2A suggest a potential role for synaptic vesicle modulation.

Additionally, accumulating evidence points to that SV2A being relatively enriched in GABAergic inhibitory neurons.^51^^,^^52^^,^^53^ Consistent with this cellular distribution, the loss of SV2A preferentially disrupts GABAergic transmission,^32^ suggesting that altered SV2A may also signify a shift in the excitatory-inhibitory balance in sleep-wake cycle. Supporting this, an increase in GABAergic spontaneous inhibitory postsynaptic currents has been observed in mice across the light phase accompanied by a decrease in dark phase under the modulation of vasoactive intestinal peptide.^54^ Bridi et al. reported that the excitatory/inhibitory (E/I) balance in regions such as the primary VC oscillates with the circadian rhythm.^55^ Furthermore, a structural study has revealed that SV2A serves as a key receptor for botulinum neurotoxin (BoNT), mediating its neuronal binding and endocytosis,^56^ which supports that SV2A expression may be associated with presynaptic metabolic state and vesicle energy regulation. Glutamate levels are elevated during wakefulness and decreased during sleep,^57^^,^^58^ suggesting an overall reduction in presynaptic (particularly glutamatergic) activity during sleep. Therefore, we propose that, despite the reduction in presynaptic activity during sleep, the elevated SV2A levels may reflect an increased total number of synaptic vesicles or vesicle release efficiency, thereby establishing functional reserves for subsequent neuronal activity during wakefulness. Conversely, during prolonged wakefulness or after sleep deprivation, sustained synaptic transmission may lead to continuous vesicle consumption, and the subsequent decrease in SV2A could stem from synaptic fatigue. Concurrently, spine pruning optimizes network architecture during sleep. Together, these processes strike a balance between presynaptic efficiency and postsynaptic structural capacity across the sleep-wake cycle. This pattern of pre- and post-synaptic regulation is echoed by a prior sleep deprivation study, which reported an increased axon-spine interface alongside a decrease in the proportion of readily releasable vesicles.^11^ Moreover, presynaptic vesicle assembly and postsynaptic density protein 95 (PSD-95) clusters were proposed to be spatiotemporally coupled,^59^ and postsynaptic density (PSD) area was found correlated with the release probability of synaptic connections.^60^ Our study provides the quantitative evidence for this trans-synaptic coordination, demonstrating coupled yet presynaptic SV2A and overall postsynaptic spine density. Furthermore, this apparent dissociation, increased spine density alongside decreased SV2A, may be mechanistically linked by the finding that spine enlargement physically pushes the presynaptic bouton and strengthens evoked release.^61^ Under this framework, the structural expansion of spines during wakefulness could drive enhanced presynaptic output, resulting in vesicle pool exhaustion reflected by reduced SV2A levels. The precise relationship between spine density and size in this mechanism remains to be explored in future studies.

Notably, a recent human [^18^F]SynVesT-1 PET study reported a 3–6% increase in SV2A binding following ∼28 h of sleep deprivation, with the magnitude of increase positively correlating with slow-wave activity during recovery sleep (Elmenhorst et al., in press). This discrepancy may partly reflect differences in sleep deprivation paradigms and associated neuronal activation states, as mice were subjected to a relatively mild gentle-handling protocol, whereas sustained wakefulness in humans typically involves continuous cognitive and sensory engagement. Importantly, these divergent findings highlight the complexity of SV2A PET signals, which may reflect not only synapse number, but also dynamic changes in synaptic vesicle pools and trafficking states that vary across species and experimental conditions. Therefore, the mechanistic framework established in the present study may provide a foundation for future cross-species investigations aimed at bridging rodent and human synaptic physiology under altered sleep-wake conditions.

In this study, the spine evidence aligns with the SHY that there is potentiation during wakefulness and down-regulation during sleep phase. We focused on the basal dendrites of L2/3 and L5 pyramidal neurons, which receive predominantly intracortical excitatory inputs and are crucial for local circuit integration. While prior research has concentrated largely on apical dendrites, our findings from a sleep-wake cycle perspective uncover a distinct aspect of postsynaptic plasticity in basal dendrites, thereby complementing the existing literature. In this study, L5 serves as the primary regulatory layer during the light phase. As the main output layer of cortical structures, L5 pyramidal neurons exhibit activity that is closely linked to behavioral states, which may render them more sensitive to circadian rhythms and light. Regarding sleep-wake-related dendritic spine density in L5 pyramidal neurons, existing evidence generally supports a higher spine elimination associated with sleep.^14^^,^^16^ However, these studies lack cross-age comparisons and direct evidence specifically targeting basal dendrites. Moreover, in adult mice, synapses have matured, establishing a stable structural foundation while retaining circadian rhythm-dependent plasticity. In contrast, adolescent mice undergo rapid brain maturation characterized by elevated synaptic density and heightened dendritic spine plasticity.^62^ As a result, photoperiod-induced changes may be partially masked by ongoing developmental remodeling.

### Long-term effects of SD on SV2A

Complementing the postsynaptic structural changes, our presynaptic [^18^F]SynVesT-1 PET imaging showed that the reduction in SV2A levels induced by 12 h of acute sleep deprivation persisted after 24 h of recovery, suggesting a slower presynaptic recovery process, contrasting with the previous rapid reversal of synaptic plasticity after milder sleep loss.^58^^,^^63^ Sleep deprivation duration critically dictates recovery length, as evidenced by hormonal studies where acute 24-h sleep loss in rats led to partially reversible changes over four days, while some hormones remained suppressed even 96 h later.^64^^,^^65^

In this study, control mice showed a non-significant SV2A decrease over three days, potentially due to repeated isoflurane exposure. While prior studies noted increased [^18^F]SynVesT-1 accumulation under anesthesia,^66^ methodological differences may account for the discrepancy. Thus, the sustained SV2A reduction in SD mice on M3 versus M2 likely stems from both incomplete synaptic recovery and cumulative anesthetic effects. Regardless, our results reveal significantly lower SV2A levels and long-term suppression in the SD group, providing longitudinal evidence of presynaptic sleep deprivation effects.

### Age-specific synaptic regulation strategies

While previous studies have reported circadian variations in synaptic density or protein levels (e.g., ∼22–30% differences between ZT0 and ZT12 in SSC^67^), few have integrated these rhythms with distinct developmental stages. Our study reveals that sleep-dependent synaptic regulation is age-specific. At two circadian time points spanning the sleep period, adolescent mice exhibited significantly higher SV2A levels and spine density than adult mice, consistent with the more active synaptogenesis and turnover during juvenile stage.^68^

Additionally, this study further reveals an age-dependent shift in sleep-related synaptic regulation. In adult individuals, SV2A expression is primarily regulated under the light, corresponding to the sleep phase. However, in young mice, SV2A is more susceptible to sleep deprivation compared to the changes across sleep phase. Although evidence on age differences in sleep plasticity remains limited, studies suggest greater synaptic plasticity in younger animals.^69^^,^^70^ For instance, 72-h deprivation robustly increased excitatory synapse density in juvenile mouse dentate gyrus, an effect absent in adults.^71^ Moreover, forebrain synapses in juvenile animals were revealed to be uniquely vulnerable to sleep deprivation.^45^ These findings align with our observations and underscore development as a key modulator of synaptic responses.

Our data further reveals an age-dependent synaptic response to prolonged wakefulness. Under identical circadian conditions, young mice exhibited sub-baseline levels of presynaptic and postsynaptic markers after sleep deprivation (SD < ZT0), reflecting their higher baseline synaptic strength and plasticity, which may limit compensatory capacity and enhance vulnerability to sleep loss. In contrast, adults, with lower baseline synaptic strength, showed elevated synaptic markers following deprivation (SD > ZT12). This likely indicates a compensatory or rebound enhancement of synaptic efficacy when synaptic saturation is not yet reached. This is supported by evidence such as sleep deprivation-induced LTP in hypocretin neurons and astrocyte-mediated modulation of excitatory transmission.^72^

This age-specific pattern aligns with reported homeostatic mechanisms: in young animals, overstimulation triggers synaptic downscaling to prevent hyperactivity, whereas in adult animals, weakened homeostasis may instead permit synaptic potentiation and increased network activity.

Thus, our study defines an age-dependent pattern of circadian synaptic regulation. In the juvenile brain, sleep facilitates structural pruning while sustaining connectivity, thus refining neural circuitry. Sleep deprivation, however, elevates metabolic demands on dense juvenile synapses, surpassing presynaptic replenishment capacity of SV2A and increasing vulnerability to dysfunction. In adults, where baseline structural connectivity is lower, sleep favors functional enhancement on presynaptic over extensive structural remodeling on postsynaptic side. Under sleep deprivation, adults show modest presynaptic reduction coupled with compensatory structural potentiation, potentially preserving network efficiency.

Our results establish age as a critical modulator of synaptic homeostasis: the developing brain is more susceptible to sleep loss, whereas the mature brain sustains, or even enhances, synaptic strength via compensatory plasticity. Future work should dissect the age-dependent dynamics of this synaptic rebound mechanism.

### Limitations of the study

However, this study has certain limitations. First, to minimize experimental interference and maintain the animals in an undisturbed state, we deliberately refrained from collecting electroencephalography (EEG) and electromyography (EMG) recordings. Consequently, direct monitoring of sleep states was not performed in this study. Instead, our sampling design was based on 12-h intervals during the light and dark phases, following well-established behavioral patterns in mice. Although individual variability in behavior may exist, this sampling interval is sufficient to capture the overall effects associated with sleep and wake phases. Future studies should incorporate non-invasive behavioral assessment methods to improve the accurate of identification of sleep and wake states and to better delineate their respective effects.

Second, dendritic spine subtypes were not classified in this study, which obscures potential regulation of specific spine populations (e.g., mushroom or thin spines) that may differentially contribute to synaptic plasticity. Additionally, the two-dimensional reconstruction of Golgi-stained neurons using light microscopy may systematically underestimate the absolute number of dendritic spines. We quantified the total number of spines on selected dendrites. This approach has certain advantages: in the absence of definitive information about which spines form functional synapses, counting all visible protrusions can serve as a general indicator of postsynaptic structural changes.

Additionally, the limited number of brain regions analyzed may not fully represent sleep-dependent synaptic changes across the cortex and related structures. Due to inherent limitations of spine visualization in other brain regions, we limited our analyses of spine density and SV2A expression to the neocortex. Although SV2A PET provides complementary longitudinal evidence for the effects of sleep deprivation at the whole-brain level, future studies with synaptic resolution are needed to elucidate the fine-dynamic mechanisms of presynaptic-postsynaptic co-regulation, such as longitudinal synaptic imaging. Addressing this will be a critical next step for future research to determine the regional specificity of this co-regulation.

Our *in vitro* approaches provide only a snapshot of synaptic properties, precluding longitudinal analysis. Future *in vivo* studies tracking dendritic spine dynamics and correlating them with presynaptic protein expression would greatly advance our understanding of trans-synaptic coordination. Specifically, integrating *in vivo* two-photon imaging with electrophysiology could resolve neurotransmitter release dynamics at single-synapse resolution and clarify the relationship between SV2A expression and spine modulation. Regarding the presynaptic compartment, SV2A as a marker has inherent limitations, as it does not specifically label excitatory or inhibitory synapse subtypes (e.g., excitatory synapses corresponding to spines). Future research using electrophysiological recordings or synapse-type-specific markers (e.g., vGluT1/vGAT imaging) to link changes in SV2A with neurotransmitter release efficiency and excitatory/inhibitory balance will further advance in-depth analysis. Additionally, this study did not provide direct evidence regarding synaptic density or transmission efficiency. Our ongoing ultrastructural analysis using electron microscopy will provide direct evidence of structural changes in the presynaptic compartment (bouton) and further clarify the morphological correlates of vesicle availability.

### Conclusion

Our study provides evidence that synaptic markers in pre- and postsynaptic compartments are regulated in a coordinated but bidirectional manner across the sleep-wake cycle. To our knowledge, this is the first demonstration that during sleep, dendritic spine pruning occurs concurrently with a global upregulation of SV2A, revealing an unexpected and dynamic form of synaptic remodeling. These findings highlight the intricate interplay between structural and molecular components of synapses, advancing our understanding of how sleep shapes synaptic function and plasticity. Importantly, our results further identify the central role of age in circadian synaptic regulation, providing key evidence for age-dependent synaptic homeostasis across the sleep-wake cycle.

## Resource availability

### Lead contact

Further information and requests for resources and reagents should be directed to and will be fulfilled by the lead contact, David Elmenhorst (d.elmenhorst@fz-juelich.de).

### Materials availability

This study did not generate new unique reagents.

### Data and code availability

• All data generated or analyzed during this study are included in this published article and its supplemental information files. Additional information is available from the lead contact upon reasonable request.
• This study did not generate custom code.
• Any additional information required to reanalyze the data reported in this paper is available from the lead contact upon reasonable request.

## Acknowledgments

This research was supported by internal institutional funds and the project SleepLess, which received funding from 10.13039/501100002347BMBF (grant no. 01EW1808), and FWO and FRQS under the frame of ERA-NET Neuron Cofund. The authors would like to thank Nikola Kornadt-Beck for excellent technical assistance. J.M. would like to thank the 10.13039/501100004543China Scholarship Council (CSC) for financial support under grant no. 202106240026.

## Author contributions

D.E., B.K., L.K.-H., and J.M. designed the study; J.M. analyzed and interpreted the data and drafted and revised the manuscript; D.E., B.K., J.M., A.B., A.O., S.D., N.H., U.H., S.J., and S.K. were responsible for experiments and data collection. D.E., B.K., and A.R. contributed to data interpretation and revision; S.S., P.K., and B.N. contributed to tracer synthesis; A.D. and A.B. contributed to revisions; S.B. supported the PET data reconstruction. All authors critically revised the manuscript and approved the final version.

## Declaration of interests

A.D. reports research support from Siemens Healthineers, Life Molecular Imaging, GE Healthcare, AVID Radiopharmaceuticals, Sofie, Eisai, Novartis/AAA, and Ariceum Therapeutics; speaker honoraria and advisory board participation from Siemens Healthineers, Sanofi, GE Healthcare, Biogen, Novo Nordisk, Invicro, Novartis/AAA, Bayer Vital, Lilly, Peer View Institute for Medical Education, International Atomic Energy Agency, and Swiss Rockets; stock ownership in Siemens Healthineers, Lantheus Holding, Lilly, and Bayer; participation in industry-sponsored trials including studies sponsored by Novartis Pharma; and a patent related to 18F-JK-PSMA-7 (patent no. EP3765097A1). A.D. also receives national and international research funding, including DFG grants (SFB 1451 C04 and DR 445/9-1) and Wellcome Leap.

## Declaration of generative AI and AI-assisted technologies in the writing process

During manuscript preparation, the authors used ChatGPT to assist with text organization and translation. All content was reviewed and edited by the authors, who take full responsibility for its accuracy and integrity.

## STAR★Methods

### Key resources table

**Table:** 

REAGENT or RESOURCE	SOURCE	IDENTIFIER
**Biological samples**		

Mice brain tissue from WT C57BL/6J mice	This study	N/A

**Chemicals, peptides, and recombinant proteins**		

Agarose	Carl Roth	Cat# 3810.3
Eukitt mounting medium	Sigma-Aldrich	Cat# 03989
[^3^H]UCB-J, Specific activity: 917.6 GBq/mmol	RC Tritec	Cat# RCTT0711
Levetiracetam	Sigma-Aldrich	Cat# PHR1447
[^18^F]SynVesT-1	This study	N/A

**Critical commercial assays**		

FD Rapid GolgiStain Kit	FD NeuroTechnologies, INC, USA	Cat# PK401

**Experimental models: Organisms/strains**		

Mouse: C57BL/6J	Charles River Laboratories	Strain Code 027

**Software and algorithms**		

Microsoft Excel	Microsoft	Microsoft 365, Version 2604
GraphPad Prism	GraphPad Software, San Diego, CA	Version 8.3.0
R	R Project for Statistical Computing	Version 4.5.1
Neurolucida	MicroBrightField Europe, Magdeburg, Germany	Version 10.0
AIDA Image Analyzer	Elysia Raytest	Version 4.13
G∗Power	Heinrich Heine University Düsseldorf	Version 3.1.9.7
PMOD	PMOD Group, Zurich, Switzerland	Version 3.408

**Other**		

Leica CM cryostat	Leica Biosystems	CM 3050 S
BAS5000 BioImage Analyzer	FUJIFILM	Fujifilm, Japan
BAS-IP TR 2025E imaging plate	Cytiva Sweden AB	Cat# 28956482
Small-animal PET scanner	Siemens	INVEON
Leica vibratome	Leica Biosystems	VT1000S

### Experimental model and study participant details

All animal experiments were approved by the German authorities (Landesamt für Natur, Umwelt und Verbraucherschutz Nordrhein-Westfalen, LANUV NRW; approval number 81–02.04.2022.A245) and conducted in accordance with German animal welfare regulations.

Wild-type C57BL/6 mice were purchased commercially (Charles River Laboratories, Sulzfeld, Germany) and were housed in groups of four with a 12:12 h light-dark at ∼22°C with humidity in 55% ± 10% with food and water provided *ad libitum*.

Animals of different developmental stages were used depending on the experimental protocol. Detailed age information for each experiment is provided in the corresponding Method Details.

Only male mice were included in this study. Therefore, the study was not designed to evaluate sex-specific effects, and the findings may not necessarily generalize to female mice.

### Method details

#### Sleep deprivation

Sleep deprivation (SD) was performed by gentle handling while minimizing the introduction of novel stimuli. Briefly, animals were monitored continuously, and whenever signs of sleep onset were observed, mice were gently stimulated by touching them with a soft paintbrush or by gently shaking the cage. These procedures were repeated throughout the SD period to maintain wakefulness.

#### Histological research

A total of 42 male C57BL/6J mice were divided into 6 experimental groups (*n* = 7 mice per group) across two age cohorts (4–6 weeks, *n* = 21; 9–24 weeks, *n* = 21). Only male mice were included to minimize potential variability arising from sex differences. The sample size was determined using G∗Power 3.1.9.7 with α = 0.05 and effect sizes derived from prior spine density studies following sleep deprivation. After at least one week of acclimation under a standardized 12/12 light-dark cycle (lights on at ZT0 and off at ZT12, with the light phase representing the natural sleep period), animals of different ages were assigned to three experimental conditions and sacrificed at three critical time points: ZT0, ZT12, and ZT12 following 12-h sleep deprivation (SD) (Figure 1A). This design, which corresponds to periods when most mice naturally consolidate sleep or wakefulness, enabled the simultaneous investigation of circadian influences and sleep deprivation effects across developmental stages. Although behavioral states (e.g., EEG or activity monitoring) were not directly recorded in this experiment, ZT0 (light onset) and ZT12 (light offset/dark onset) were used as reference points, serving as reasonable proxies for periods of sleep -like tendencies, respectively.

Brain tissues were collected immediately after euthanasia. Each brain was sagittal sectioned along the midline to separate the two hemispheres. The left hemisphere was fixed for Golgi impregnation, and the right hemisphere was processed for autoradiography.

#### Golgi impregnation

The Golgi-COX kit used in this research was purchased commercially (FD Rapid GolgiStain Kit, FD NeuroTechnologies, INC, USA). In detail, brain tissues were extracted from animals, immediately rinsed with double distilled water (ddH_2_O), then immersed in impregnation solution 1 overnight at room temperature. The next day, tissue samples were incubated in fresh impregnation solution 1 and stored for 14 days in the dark at room temperature. Subsequently, the tissue was transferred into solution 3 and kept in the dark at room temperature for one day. Thereafter, it was placed into fresh solution 3 and kept in the dark at room temperature for 6 additional days. Then, solution 3 was exchanged and samples were stored at 4°C in the dark overnight. The tissue container holding the tissue was gently swirled side-to-side for a few seconds twice a week during the impregnation period. After the impregnation, brain tissues were embedded in 4%–5% agarose (Carl Roth, Karlsruhe, Germany) in H_2_O and cut into 200-μm sections using a vibratome (VT 1000S; Leica Biosystems GmbH, Wetzlar, Germany) and transferred to ddH_2_O. After removal of the agarose, free-floating sections were incubated into solution 3 for 2–3 min in the dark at room temperature, and right after placed into ddH_2_O, washed several times and stored overnight. Afterward, they were rinsed twice in ddH_2_O for 4 min each, and dehydrated in 50%, 70% and 95% ethanol for 5 min each, then transferred into absolute ethanol (3 × 5 min), defatted in xylene, embedded in Eukitt (Sigma-Aldrich Chemie GmbH, Taufkirchen, Germany), finally coverslipped and air-dried.

Neurons were observed under a microscope (Olympus, Hamburg, Germany) using the following objectives: 4× (UPlanApo, NA 0.16); 10× (UPlanApo, NA 0.40); 60× (PlanApo Oil, NA 1.40); 100× (PlanApo Oil, NA 1.40). Dendritic spines were quantified using Neurolucida (MicroBrightfield Europe, Magdeburg, Germany) at 60× or 100× magnification. The somatosensory cortex (SSC) and visual cortex (VC) were delineated based on anatomical location, with the hippocampus serving as a reference. Pyramidal neurons in layers 2/3 and 5 were identified by their characteristic morphology and laminar position and the spine density per 1 μm dendritic length of basal dendrites was calculated (Figure 1B). A total of over 1500 dendritic segments were reconstructed in this research (see Table S2). Only clearly visible dendrites longer than 20 μm were included in the analysis. The dendrites analyzed in this study had a mean length of 107.1 ± 73.8 μm (mean ± SD).

#### Autoradiography

Following extraction, brain samples were immediately frozen in chilled isopentane and subsequently stored at −80°C until processing. Using a Leica CM cryostat (Leica Biosystems) maintained at −20°C, 20 μm sagittal sections were cut and thaw-mounted onto adhesive silica-coated microscope slides (StarFrost). [^3^H]UCB-J (specific activity = 917.6 GBq/mmol) was obtained commercially (RC Tritec AG, Switzerland). 4 slices of each animal were used for autoradiography, 3 slides for total binding incubation and one for non-specific binding assay. All slices, despite being for total binding or non-specific binding, were first incubated in 50 mM Tris-HCl buffer (pH 7.4) for 10 min at room temperature (RT) for pre-incubation. Sections for total binding were subsequently incubated with [^3^H]UCB-J (9.56 nM) in 50 mM Tris-HCl buffer containing 5 mM MgCl_2_ (pH 7.4) for 2 h at RT. Nonspecific-binding determination were conducted using the same [^3^H]UCB-J solution supplemented with 1 mM levetiracetam (Sigma-Aldrich). Table S1 presents incubation procedures of slices.

After incubation, all slides were air-dried and exposed to a BAS-IP TR 2025E imaging plate (Cytiva Sweden AB) for 3 days at RT with tritium activity standards. Plates were scanned using a high-performance plate reader (spatial resolution of 50 μm; BAS5000 BioImage Analyzer, Raytest-Fuji, Straubenhardt, Germany), and images were acquired for analysis. [^3^H]UCB-J binding was quantified using AIDA Image Analyzer (v.4.13). Signal intensity was converted to fmol/mg protein using a calibration curve. Data were adjusted by a correction factor (α), calculated as: α = K_d_/F + 1 (K_d_ = 34 nM, F = 9.56 nM).

#### [^18^F] SynVesT-1 PET imaging

A total of 14 male mice (C57BL/6J, 9–16 weeks) were included for sleep deprivation study with [^18^F]SynVesT-1 PET imaging. Animals were housed in the local facility under an inverted 12/12 h light/dark circle in a shielded light box with ventilation (light-on: 10 PM-10 AM, light-off: 10 AM-10 PM). Longitudinal PET imaging was performed over three consecutive days, with all scans performed at 10 AM. On the first day, animals were scanned at the end of the light phase (ZT24), corresponding to the end of the natural sleep period (after sleep, AS). Mice were then sleep deprived by gentle handling until the second scanning at ZT48 (after sleep deprivation, SD). Afterward, animals were left in their home cages without further disruption and scanned again at ZT72 following one day of recovery from sleep deprivation (R) (Figure 4A). The control group (*n* = 7, 13–15 weeks, male) underwent the same three-day scanning schedule at the end of light on phase but without any disturbance. (Figure 4B).

#### Radiochemistry

[^18^F] SynVesT-1 was formulated and synthesized as follows: The fully GMP-compliant automated radiosynthesis of [^18^F]SynVesT-1 was performed using the Trasis AllinOne (AIO) synthesizer. The process began with the transfer and trapping of approximately 37 GBq of [^18^F] fluoride on a QMA cartridge. The reactor was initially heated to 65 °C. Subsequently, a syringe was prepared containing 2 mg of tetraethylammonium bicarbonate in 1 mL of methanol along with 0.8 mL of air. This mixture was then used to elute the [^18^F] fluoride into the reactor. The reactor temperature was increased to 80 °C, followed by a drying phase consisting of three steps under vacuum with nitrogen: 80 s at 115 °C, 180 s at 125 °C, and 124 s at 95 °C. After drying, the reactor was cooled to 55 °C, and 5 mg of precursor along with 20 mg of tetrakis(pyridine) copper(II) triflate in 0.8 mL of N,N-dimethylacetamide (DMA) were added. The 18F-fluorination reaction proceeded at 110 °C for 20 min. After reaction, the reactor was cooled to 40 °C. The resulting mixture was purified by reversed-phase high-performance liquid chromatography (RP-HPLC) using a Phenomenex Luna 5μm C18 column (100 Å, 250 × 10 mm). The mobile phase consisted of an acetonitrile/water mixture (1:3) with 5.4 g of ammonium acetate (NH_4_Ac) at pH 4.4. Further purification was achieved through solid-phase extraction (SPE) using a C18 cartridge, with elution performed using ethanol and washing with a 0.9% saline solution. The preparation of [^18^F]SynVesT-1 resulted in a radiochemical yield (rcy) of up to 20% and a radiochemical purity of 99%. The radiotracer was diluted with sterile saline solution (0.9%) and injected as intravenous bolus. The [^18^F]SynVesT-1 had a molar activity of 50 ± 32 GBq/μmol.

#### Acquisition of [^18^F]SynVesT-1 PET images

Anesthesia was induced with 5% isoflurane in 2 L/min oxygen and then maintained with 1–2% isoflurane in 2 L/min oxygen, during which animals were placed on a heating pad set to 39°C. A tail vein catheter (PE10, Becton Dickinson, Sparks, MD, USA) filled with heparinized physiological saline solutions was applied to mice. Simultaneously, respiratory rate and body temperature were recorded. During imaging acquisition, pressure-sensitive pads were placed under the mice’s chest to record the respiration (BioVet System, m2m Imaging, Salisbury, QLD, Australia). The temperature was kept stable with a heating light monitored by a rectal probe. PET images were acquired using a Siemens Inveon scanner (Siemens, Knoxville, TN, USA) with Inveon Acquisition Workplace (IAW) 1.5 (Siemens) under anesthesia with isoflurane following the intravenous bolus injection of [^18^F]SynVesT-1 (6.01 ± 0.98 MBq) in approximately 150 μL of saline in 2 min. Dynamic PET images were reconstructed into 33 frames of varying lengths (12 × 10 s, 3 × 20 s, 3 × 30 s, 3 × 60 s, 3 × 150 s, and 9 × 300 s).

#### Image processing and modeling with image-derived input function (IDIF)

PET image processing was performed using PMOD software (version 3.408, PMOD Group, Zurich, Switzerland). Mouse brains were first co-registered to the Mirrione-T2 mouse brain atlas. Time-activity curves of PET images were extracted from regions of interest in the template, including the striatum, cortex, hippocampus, thalamus, and cerebellum. Regional time-activity curves were normalized to the corresponding injected dose and body mass to obtain the standardized uptake value (SUV, g/mL) time curve on PMOD. To compare the difference in uptake of tracer between groups, the standard uptake values of 30-60 mins time points were averaged to calculate the adjusted SUV (aSUV).

For kinetic modeling of PET images, an image-derived input function (IDIF) was measured in the heart by placing a 10 mm cubic volume of interest (VOI) over the heart and thresholding voxels within the VOI that exceeded 50% of the total activity. Noninvasive IDIF has recently been demonstrated as an effective method to quantify [^18^F]SynVesT-1 binding in rats.^73^ The population-based metabolite and plasma/whole blood ratio from this publication was used to correct the IDIF and scaled with a predetermined factor α = 0.77 to account for partial volume effects of the specific PET scanner.^74^

Specifically, the one-tissue compartment model (1TCM), two tissue compartment model (2TCM) and Logan plot were used for kinetic modeling with image-derived input function (IDIF) on PMOD 3.408. For 1TCM and 2TCM, the blood volume fraction (V_B_) was fixed at 3.6% as provided by ref.^73^ Total distribution volume (V_T_, mL/cm^3^) from different models were obtained from IDIF.

### Quantification and statistical analysis

All statistical analyses were performed using Microsoft Excel, GraphPad Prism 8.3.0 (GraphPad Software, San Diego, CA) or R (version 4.5.1). Data normality was assessed using the Shapiro-Wilk test (α = 0.05). All data were analyzed with linear mixed-effects models fitted with restricted maximum likelihood (REML), with animal ID included as a random intercept to account for intra-animal clustering. The models assessed the effects of sleep condition (ZT0, ZT12, sleep deprivation), age cohort, and brain region on dendritic spine density and [^3^H]UCB-J binding. Degrees of freedom were estimated using the Kenward-Roger method, and *t*-tests were performed using the Satterthwaite approximation. For multiple comparisons, confidence intervals and *p*-values were adjusted using the Sidak method. Statistical significance was set at *p* < 0.05. Additional details of the analyses are provided in the supplemental information. Data are presented as mean ± SEM unless otherwise stated. Significance levels are indicated as follows: ∗*p* < 0.05, ∗∗*p* < 0.01, ∗∗∗*p* < 0.001, and ∗∗∗∗*p* < 0.0001.

#### Autoradiography data analysis

Specific binding was quantified in the neocortex and hippocampus. Within the neocortex, the somatosensory cortex (SSC) and VC were further delineated, and layer 2/3 and layer 5 within these regions were then identified and analyzed separately. Specific binding of [^3^H]UCB-J was analyzed with linear mixed-effects models. For comparisons between neocortex and hippocampus, data were analyzed with the model: *Specific Binding ∼ Group × Age × Region* + *(1 | Animal).* For subregion-level comparisons (somatosensory cortex and visual cortex), region was treated as the subregion factor and modeled as: *Specific Binding ∼ Group × Age × Region + (1 | Animal).* For SSC and VC, where layer-specific data (L2/3 and L5) were available, a focused analysis including layer was performed: *Specific Binding ∼ Group × Age × Layer × Region + (1 | Animal ID)*.

#### Spine density analysis

Technical limitations in the Golgi staining process resulted in an incomplete and variable dataset across the planned cohorts, as not all cortical areas were sampled in every animal, and the yield of quantifiable dendritic segments was inconsistent. Therefore, we analyzed spine density using a linear mixed-effects model, fitted by restricted maximum likelihood (REML), with animal IDs included as the random intercept to account for intra-animal clustering effects. The fixed-effects structure was set as: *Spine density ∼ Group × Age × Layer × Region + (1 | Animal ID)*.

#### PET data analysis

PET-derived V_T_ values were analyzed separately for each brain region using a linear mixed-effects model with the fixed-effects structure: *V*_*T*_ *∼ Group × Measurement + (1 | Animal ID)*. For each region, pairwise comparisons were performed to assess changes across 3 scans within each group and to compare groups at each scan.
