# Supplementary material for: Sleep differentially modulates synaptic vesicle protein 2A and spine density across cortical regions and development
Source: iScience. 2026 Jul 15;29(8):116676. doi: 10.1016/j.isci.2026.116676 (PMC13383866; doi:10.1016/j.isci.2026.116676)
Supplement: Document S1. Figures S1–S10 and Tables S1–S5 [file mmc1.pdf]

iScience, Volume *29*

Supplemental information

Sleep differentially modulates synaptic vesicle protein 2A and spine density across cortical regions and development

Jing Ma, Alexandra Braun, Angela Oskamp, Lena Kricsfalussy-Hrabár, Nadja Hermes, Salma Djakkani, Ulrike Holz, Sabine Jakobs, Sabina Klein, Stefan Stüsgen, Philipp Krapf, Bernd Neumaier, Alexander Drzezga, Simone Beer, Andreas Bauer, Astrid Rollenhagen, Björn Kampa, and David Elmenhorst

Supplemental Information

Table S1. The procedures for [^{3}H]UCB-J autoradiography were performed according to the steps outlined below. Triplicate sections were used for total binding incubation, and one section was used for non-specific binding incubation.

Procedure	Solution	Duration and Temperature
Pre-incubation	50 mM Tris-HCl (pH 7.4)	10 min, RT
Main incubation	Total binding:<br>50 mM Tris-HCl (pH 7.4)<br>+ 5 mM $MgCl_2$<br>+ [^{3}H]UCB-J (9.56 nM)<br>Non-specific binding:<br>50 mM Tris-HCl (pH 7.4)<br>+ 5 mM $MgCl_2$<br>+ [^{3}H]UCB-J (9.56 nM)<br>+ 1 mM levetiracetam	120 min, 4 ℃
Washing 1	50 mM Tris-HCl (pH 7.4)	2 × 10 min, 4 ℃
Washing 2	Distilled water	1 × dip, 4 ℃
Drying		RT

Table S2. The number of reconstructed dendrites for each experimental group is listed. Pyramidal neurons in layer 2/3 (L2/3) and layer 5 (L5) of the somatosensory cortex (SSC) and visual cortex (VC) were identified, and spines on their basal dendrites were counted. Mice brain tissues were obtained at ZT0, ZT12 and ZT12 following sleep deprivation (SD). The number of animals (N) and dendrites (n) analyzed in each group is indicated.

Region	Group	Age	Layer	n (dendrite)	N (animal)
SSC	ZT0	adult	L2/3	76	4
			L5	70	4
		adolescent	L2/3	83	4
			L5	73	4
	ZT12	adult	L2/3	58	5
			L5	45	4
		adolescent	L2/3	79	5
			L5	78	5
	ZT12(SD)	adult	L2/3	79	4
			L5	71	4
		adolescent	L2/3	50	4
			L5	51	4
VC	ZT0	adult	L2/3	67	3
			L5	46	3
		adolescent	L2/3	64	2
			L5	63	2
	ZT12	adult	L2/3	77	5
			L5	48	4
		adolescent	L2/3	97	4
			L5	59	4
	ZT12(SD)	adult	L2/3	87	4
			L5	54	4
		adolescent	L2/3	39	3
			L5	42	3

Table S3. Spine density (spines/μm) in the SSC and VC regions of mice measured at ZT0, ZT12, and following sleep deprivation. Density is quantified as spines per micrometer of dendrite length. Percentage change was calculated for two comparisons: ZT12 relative to ZT0, $\%Change = (Mean_{(ZT12)} - Mean_{(ZT0)}) / Mean_{(ZT0)} \times 100\%$; Sleep Deprivation (SD) relative to ZT12, $\%Change = (Mean_{(SD)} - Mean_{(ZT12)}) / Mean_{(ZT12)} \times 100\%$. Net changes in spine density (spines/μm) are shown as $Mean_{(ZT12)} - Mean_{(ZT0)}$ or $Mean_{(SD)} - Mean_{(ZT12)}$, respectively. Values are presented as mean ± SEM.

**Adult**	**SSC**			**VC**		
	**ZT0**	**ZT12**	**SD**	**ZT0**	**ZT12**	**SD**
Spine density L2/3	0.48±0.03	0.51±0.05	0.59±0.03	0.50±0.02	0.40±0.03	0.57±0.02
Net change		0.033	0.077		-0.099	0.166
Percentage of change		6.9%	+15.1%		-19.8%	+41.5%
Spine density L5	0.43±0.03	0.30±0.02	0.50±0.02	0.49±0.03	0.30±0.02	0.45±0.03
Net change		-0.129	0.205		-0.196	0.155
Percentage of change		-30.2%	+68.5%		-40.0%	+52.2%
**Adolescent**	**SSC**			**VC**		
	**ZT0**	**ZT12**	**SD**	**ZT0**	**ZT12**	**SD**
Spine density L2/3	0.63±0.02	0.61±0.02	0.70±0.03	0.67±0.03	0.52±0.03	0.59±0.02
Net change		-0.019	0.082		-0.142	0.065
Percentage of change		-3.0%	+13.4%		-21.3%	+12.3%
Spine density L5	0.68±0.02	0.60±0.02	0.60±0.02	0.61±0.03	0.53±0.02	0.62±0.02
Net change		-0.082	-0.020		-0.082	0.086
Percentage of change		-12.1%	-3.3%		-13.3%	+16.1%

Table S4. SV2A expression (pmol/mg protein) quantified by [^{3}H]UCB-J autoradiography in the somatosensory (SSC) and visual (VC) cortices of adult and adolescent mice under different sleep conditions. Percentage change was calculated for two comparisons: ZT12 relative to ZT0, $\%Change = (Mean_{(ZT12)} - Mean_{(ZT0)}) / Mean_{(ZT0)} \times 100\%$; Sleep Deprivation (SD) relative to ZT12, $\%Change = (Mean_{(SD)} - Mean_{(ZT12)}) / Mean_{(ZT12)} \times 100\%$. Net changes in SV2A levels (pmol/mg protein) are shown as $Mean_{(ZT12)} - Mean_{(ZT0)}$ or $Mean_{(SD)} - Mean_{(ZT12)}$, respectively. Values are presented as mean ± SEM.

**Adult**	**SSC**			**VC**		
	**ZT0**	**ZT12**	**SD**	**ZT0**	**ZT12**	**SD**
Entire SSC	33.07 ± 2.84	41.91 ± 1.05	37.94 ± 1.31	31.08 ± 2.32	40.40 ± 1.35	35.85 ± 1.50
Net change	8.85		-3.24	9.32		-4.55
Percentage of change	26.8%		-7.7%	30.0%		-11.3%
Layer 2/3	34.60 ± 2.42	42.49 ± 1.28	41.26 ± 1.48	31.49 ± 2.23	37.83 ± 1.79	34.94 ± 1.42
Net change	7.89		-1.23	6.34		-2.89
Percentage of change	22.8%		-2.9%	20.1%		-7.6%
Layer 5	33.87 ± 2.74	45.82 ± 1.06	40.48 ± 1.05	32.35 ± 2.60	42.52 ± 1.23	40.48 ± 1.63
Net change	11.96		-5.35	10.16		-2.04
Percentage of change	35.3%		-11.7%	31.4%		-4.8%

**Adolescent**	**SSC**			**VC**		
	**ZT0**	**ZT12**	**SD**	**ZT0**	**ZT12**	**SD**
Entire VC	43.90 ± 1.28	46.42 ± 1.77	41.38 ± 1.87	45.40 ± 2.00	45.22 ± 2.65	34.37 ± 1.50
Net change	2.52		-5.04	-0.18		-10.85
Percentage of change	5.7%		-10.9%	-0.4%		-24.0%
Layer 2/3	46.12 ± 2.10	46.86 ± 2.08	41.92 ± 1.95	44.84 ± 2.85	45.75 ± 2.27	33.81 ± 1.89
Net change	0.75		-4.95	0.91		-11.94
Percentage of change	-1.6%		-10.6%	-2.0%		-26.1%
Layer 5	45.87 ± 1.59	49.16 ± 1.73	44.99 ± 2.15	44.96 ± 2.48	51.67 ± 3.82	38.98 ± 1.25
Net change	3.29		-4.17	6.70		-12.69
Percentage of change	7.2%		-8.5%	14.9%		-24.6%

Table S5. Volume of distribution (mL/cm^3) derived from image-derived input function (IDIF) for baseline [^{18}F]SynVesT-1 PET imaging in mice (n = 14, males, 10-16 weeks), as quantified by the one-tissue compartment model (1TCM), two-tissue compartment model (2TCM), and Logan plot.

Region	$V_{T(IDIF)}$ 1TCM (mL/cm^3)		$V_{T(IDIF)}$ 2TCM (mL/cm^3)		$V_{T(IDIF)}$ Logan plot (mL/cm^3)	
	Mean ± SD	%COV	Mean ± SD	%COV	Mean ± SD	%COV
STR	28.79 ± 4.39	15.25%	29.46 ± 4.59	15.58%	26.51 ± 3.70	13.96%
CTX	23.42 ± 3.75	16.01%	24.26 ± 4.08	16.82%	22.02 ± 3.34	15.15%
HIP	29.98 ± 4.57	15.23%	31.00 ± 4.82	15.54%	28.08 ± 3.92	13.95%
THA	28.29 ± 4.38	15.49%	29.73 ± 4.82	16.22%	27.22 ± 3.87	14.21%
CB	22.93 ± 3.51	15.31%	24.02 ± 3.74	15.58%	22.12 ± 3.13	14.14%
BF	24.66 ± 3.83	15.55%	26.14 ± 3.99	15.27%	23.34 ± 3.37	14.45%
HPT	24.25 ± 3.79	15.65%	26.32 ± 4.07	15.45%	23.64 ± 3.52	14.91%
AMG	21.97 ± 3.49	15.89%	23.63 ± 3.59	15.18%	20.77 ± 2.93	14.12%
BS	18.89 ± 2.83	14.95%	20.38 ± 2.89	14.20%	19.25 ± 2.73	14.17%
OB	20.70 ± 3.93	18.99%	23.05 ± 4.16	18.03%	20.55 ± 3.51	17.08%
MB	27.01 ± 3.90	14.43%	28.60 ± 3.86	13.49%	26.61 ± 3.65	13.71%

Supplementary Figures

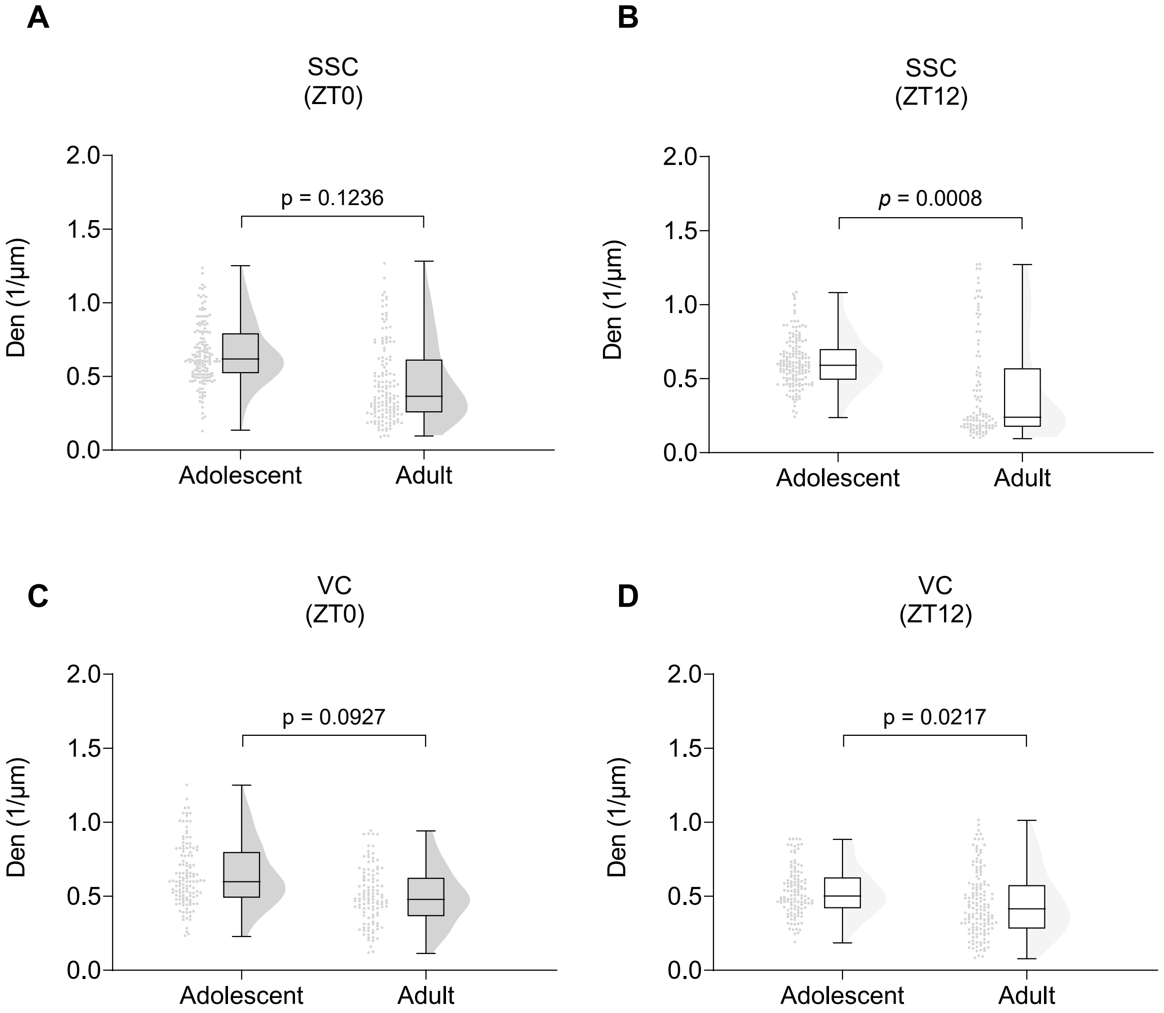

Fig S1. Comparisons of spine density (spines/μm) between adolescent (4-6 weeks) and adult (9-24 weeks) mice in the (A, B) somatosensory cortex (SSC) and (C, D) visual cortex (VC) measured at ZT0 and ZT12. Each data point represents the spine density of a single dendrite. Statistical significance was assessed using linear mixed-effects models (REML), with Sidak-adjusted post hoc comparisons. Degrees of freedom were estimated using the Kenward-Roger method, and Satterthwaite's approximation was used for *t*-tests. Significance levels are indicated as follows: * $p < 0.05$, ** $p < 0.01$, *** $p < 0.001$, and **** $p < 0.0001$. Data are represented as mean ± SEM.

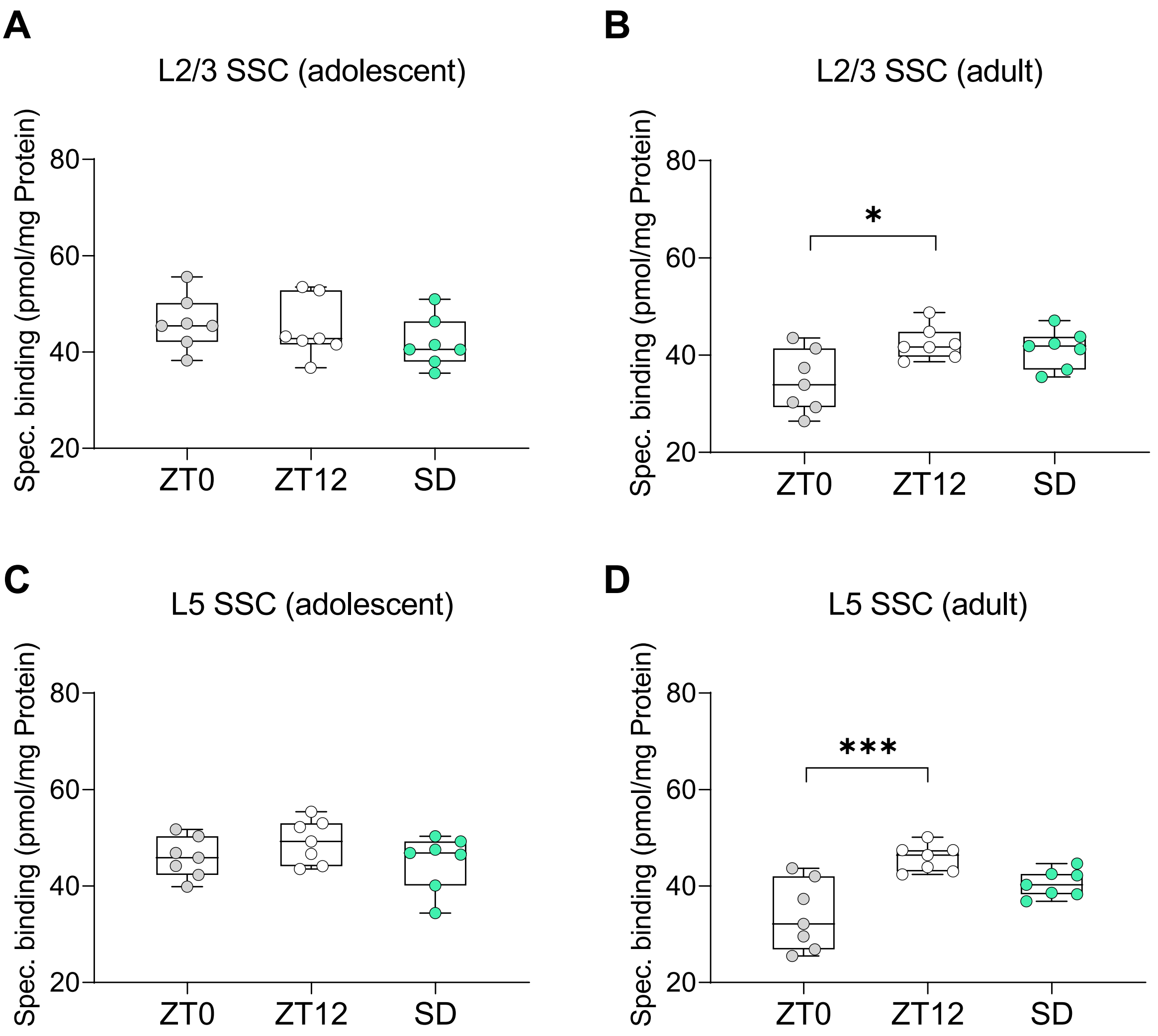

Fig S2. Specific binding of [^{3}H]UCB-J (pmol/mg protein) in (A, B) layer 2/3 (L2/3) or (C, D) layer 5 (L5) of somatosensory cortex (SSC) of adult mice (9-24 weeks) and adolescent mice (4-6 weeks) measured ZT0, ZT12, and after sleep deprivation (SD). Each dot corresponds to an individual animal. Statistical significance was assessed using linear mixed-effects models (REML), with Sidak-adjusted post hoc comparisons. Degrees of freedom were estimated using the Kenward-Roger method, and Satterthwaite's approximation was used for *t*-tests. Significance levels are indicated as follows: * $p < 0.05$, ** $p < 0.01$, *** $p < 0.001$, and **** $p < 0.0001$. Data are represented as mean ± SEM.

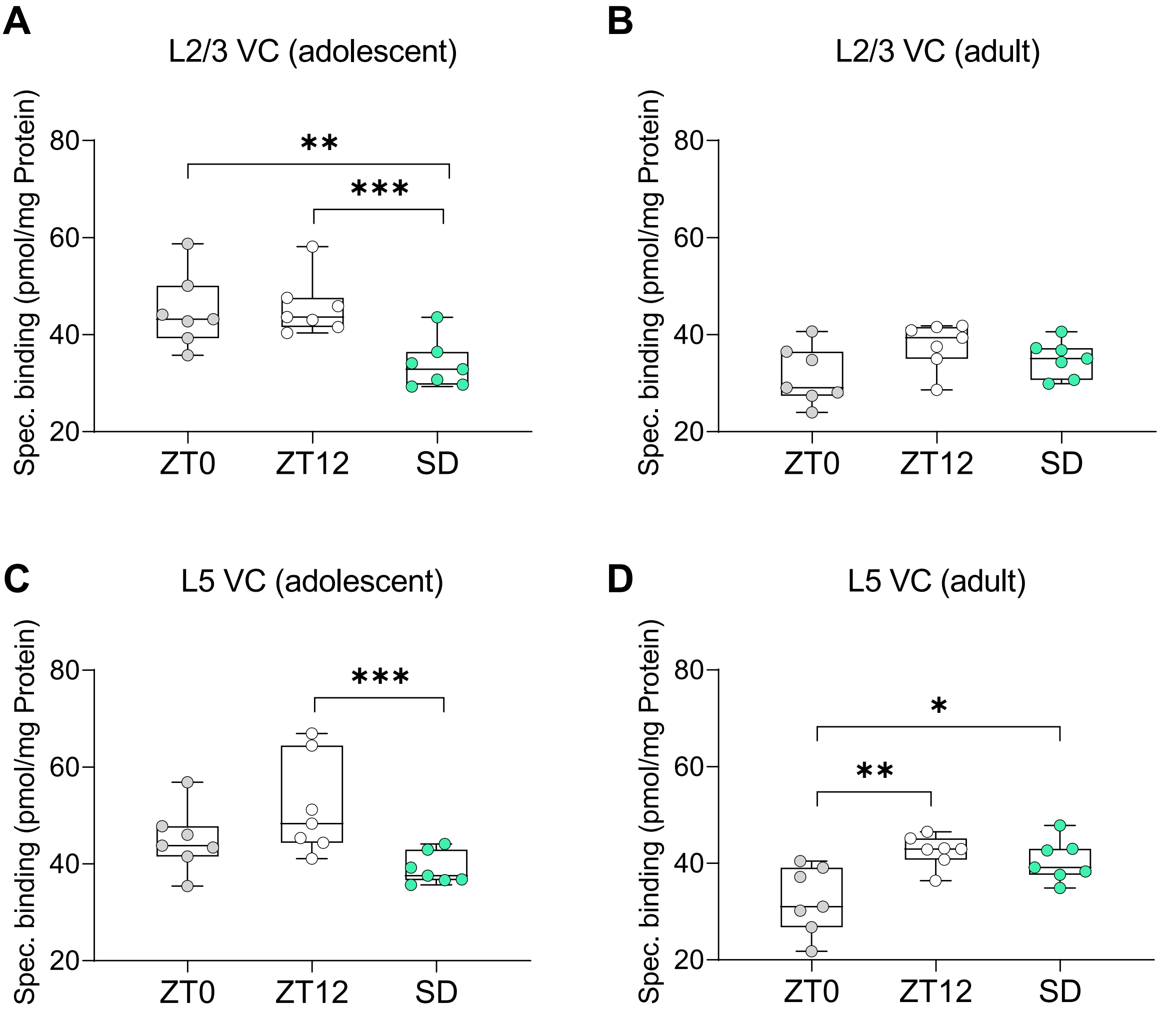

Fig S3. Specific binding of $[^3H]$UCB-J (pmol/mg protein) in (A, B) layer 2/3 (L2/3) or (C, D) layer 5 (L5) of visual cortex (VC) of adult mice (9-24 weeks) and adolescent mice (4-6 weeks) measured ZT0, ZT12, and after sleep deprivation (SD). Each dot corresponds to an individual animal. Statistical significance was assessed using linear mixed-effects models (REML), with Sidak-adjusted post hoc comparisons. Degrees of freedom were estimated using the Kenward-Roger method, and Satterthwaite's approximation was used for *t*-tests. Significance levels are indicated as follows: * $p < 0.05$, ** $p < 0.01$, *** $p < 0.001$, and **** $p < 0.0001$. Data are represented as mean ± SEM.

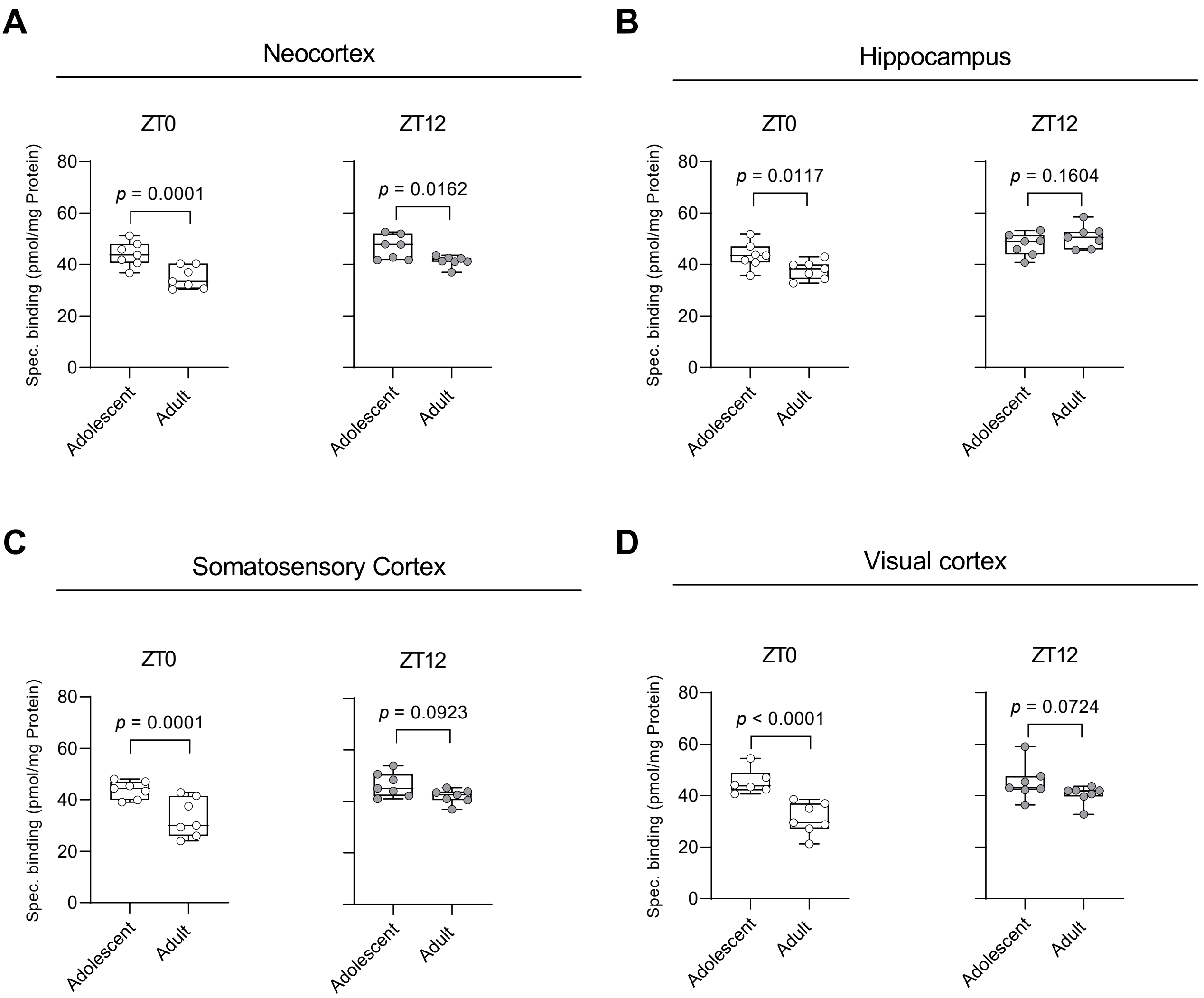

Fig S4. Age-related difference in [^{3}H]UCB-J specific binding (pmol/mg protein) between adolescent (n=7, males, 4-6 weeks old) and adult mice (n=7, males, 9-24 weeks old) measured in the (A) neocortex, (B) hippocampus, (C) somatosensory cortex and (D) visual cortex at ZT0 and ZT12. Each dot corresponds to an individual animal. Statistical significance was assessed using linear mixed-effects models (REML), with Sidak-adjusted post hoc comparisons. Degrees of freedom were estimated using the Kenward-Roger method, and Satterthwaite's approximation was used for *t*-tests. Significance levels are indicated as follows: * $p < 0.05$, ** $p < 0.01$, *** $p < 0.001$, and **** $p < 0.0001$. Data are represented as mean ± SEM.

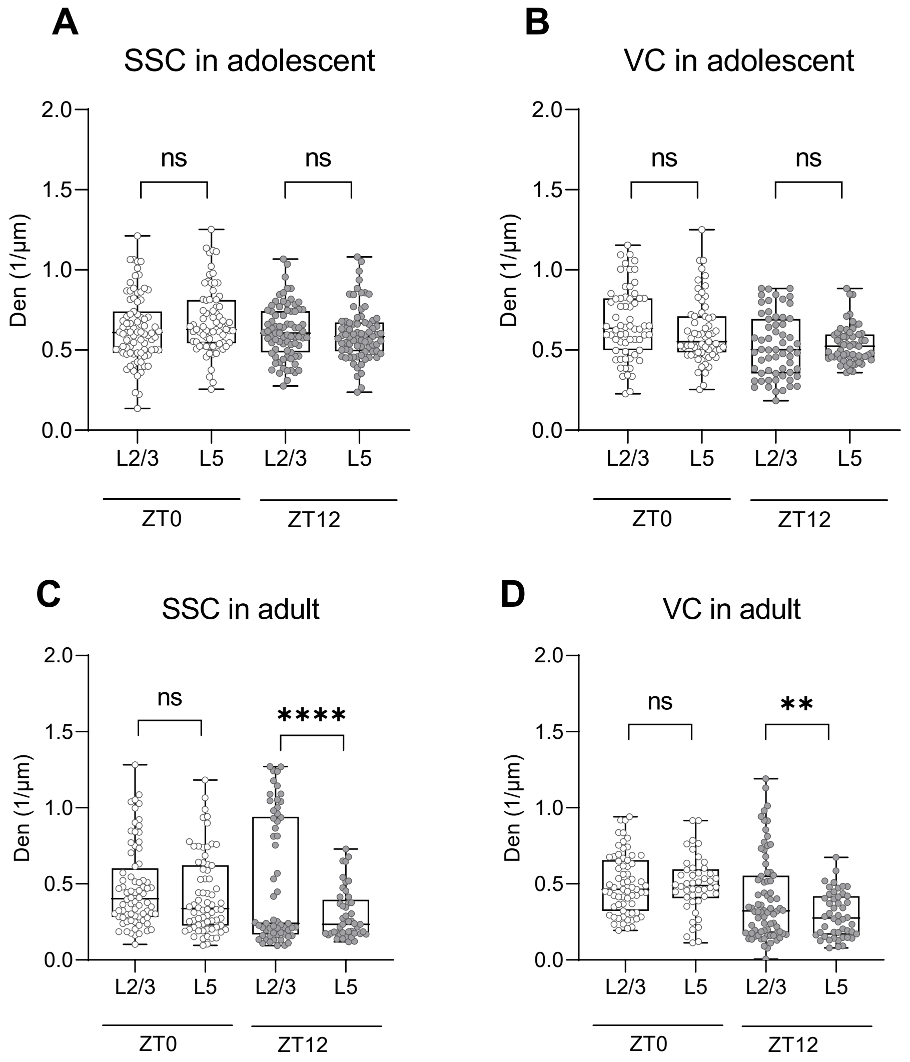

Fig S5. Comparisons of spine density (spines/μm) in layer 2/3 (L2/3) and layer 5 (L5) of the (A, C) somatosensory cortex and (B, D) visual cortex in adolescent (4-6 weeks) and adult (9-24 weeks) mice, measured at ZT0 and ZT12. Each data point represents the spine density of a single dendrite. Statistical significance was assessed using linear mixed-effects models (REML), with Sidak-adjusted post hoc comparisons. Degrees of freedom were estimated using the Kenward-Roger method, and Satterthwaite's approximation was used for *t*-tests. Significance levels are indicated as follows: * $p < 0.05$, ** $p < 0.01$, *** $p < 0.001$, and **** $p < 0.0001$. Data are represented as mean ± SEM.

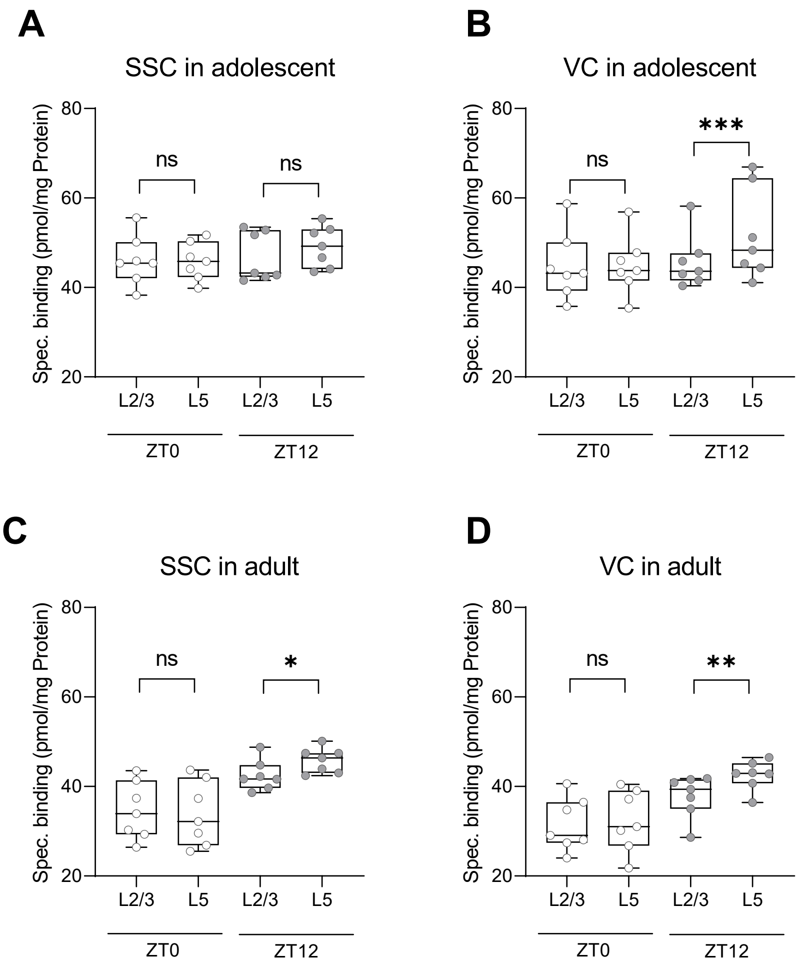

Fig S6. Comparisons of specific binding of [^{3}H]UCB-J (pmol/mg protein) in layer 2/3 and layer 5 of the (A, C) somatosensory cortex and (B, D) visual cortex in adolescent (4-6 weeks) and adult (9-24 weeks) mice, measured at ZT0 and ZT12. Each dot corresponds to an individual animal. Statistical significance was assessed using linear mixed-effects models (REML), with Sidak-adjusted post hoc comparisons. Degrees of freedom were estimated using the Kenward-Roger method, and Satterthwaite's approximation was used for *t*-tests. Significance levels are indicated as follows: * $p < 0.05$, ** $p < 0.01$, *** $p < 0.001$, and **** $p < 0.0001$. Data are represented as mean ± SEM.

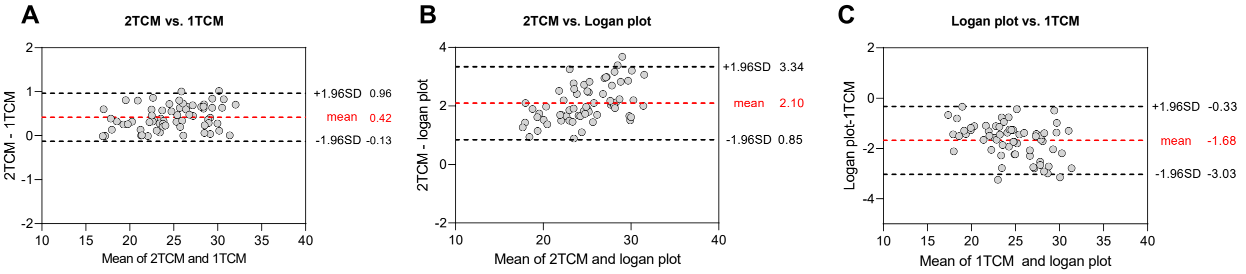

Fig. S7 Bland-Altman analysis for comparison of V_T estimation methods. V_T values were derived from image-derived input function (IDIF) for baseline [^{18}F]SynVesT-1 PET imaging in mice (n = 14, males, 10-16 weeks old) using one-tissue compartment model (1TCM), two-tissue compartment model (2TCM), and Logan plot.

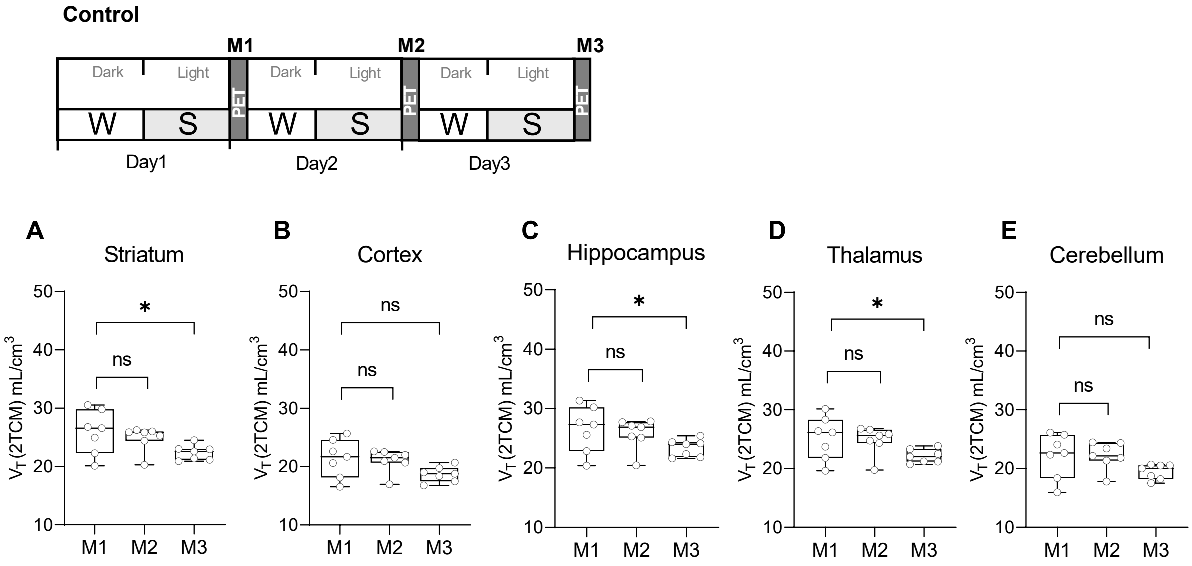

Fig S8. The total distribution volume ($V_{T(2TCM)}$, mL/cm^3) of [^{18}F]SynVesT-1 in the (A) striatum, (B) cortex, (C) hippocampus, (D) thalamus, and (E) cerebellum of control group (n = 7, male, 13-15 weeks) measured at the end of the sleep phase on three consecutive days (measurement 1, M1; measurement 2, M2; measurement 3, M3) without experimental intervention. Each dot corresponds to an individual animal. Statistical significance was assessed using linear mixed-effects models (REML), with Sidak-adjusted post hoc comparisons. Degrees of freedom were estimated using the Kenward-Roger method, and Satterthwaite's approximation was used for *t*-tests. Significance levels are indicated as follows: * $p < 0.05$, ** $p < 0.01$, *** $p < 0.001$, and **** $p < 0.0001$. Data are represented as mean ± SEM.

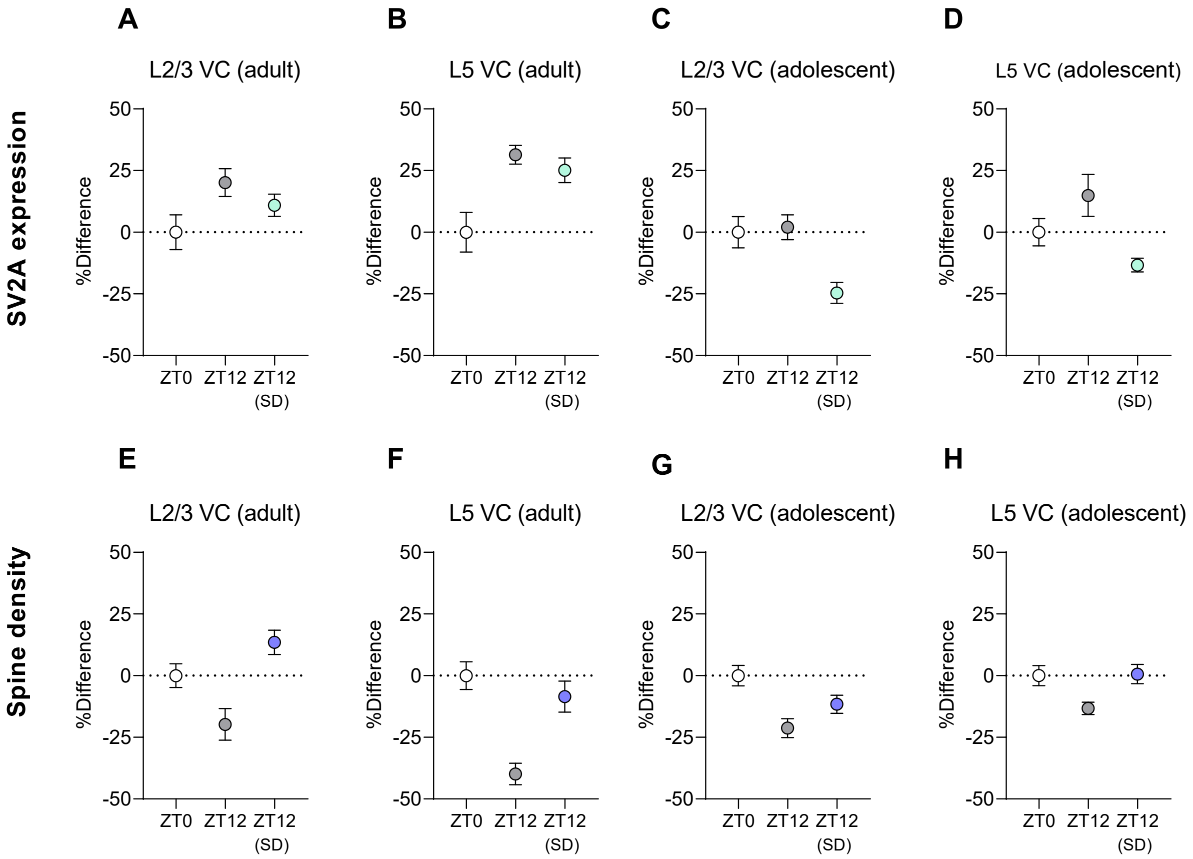

Figure S9. Normalized changes in presynaptic SV2A expression and postsynaptic spine density across sleep-wake conditions in the visual cortex of adolescent and adult mice. (A–D) Normalized presynaptic SV2A expression levels quantified by $[^3H]$UCB-J autoradiographic specific binding in layer 2/3 and layer 5 of the visual cortex (VC) in adolescent and adult mice at ZT0, ZT12, and ZT12 following sleep deprivation (SD). (E–H) Normalized postsynaptic spine density in layer 2/3 and layer 5 of the visual cortex (VC) in adolescent and adult mice at ZT0, ZT12, and ZT12 following sleep deprivation (SD). Normalization was calculated relative to the mean value at ZT0 using the formula: $\%Difference_{(i)} = (value_{(i)} - mean_{(ZT0)}) / mean_{(ZT0)} * 100\%$. Data are represented as mean ± SEM.

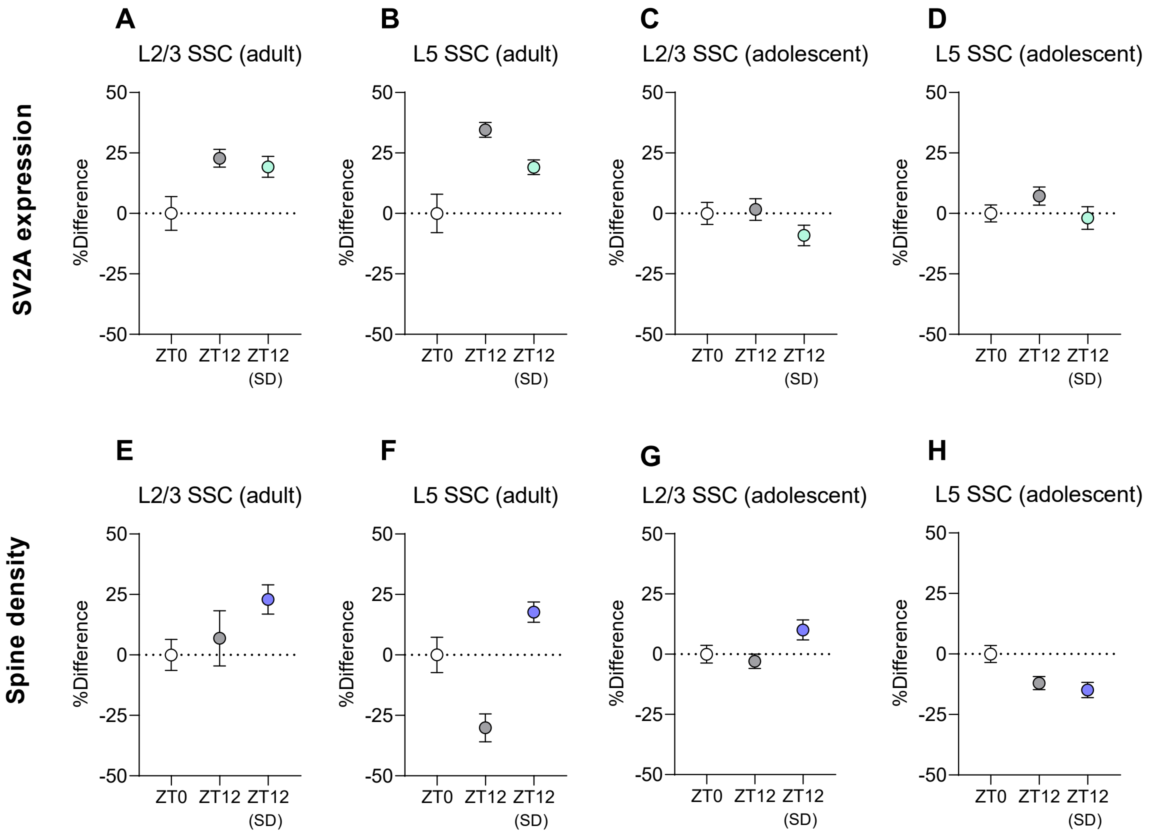

Figure S10. Normalized changes in presynaptic SV2A expression and postsynaptic spine density across sleep-wake conditions in the somatosensory cortex of adolescent and adult mice. (A–D) Normalized presynaptic SV2A expression levels quantified by [^{3}H]UCB-J autoradiographic specific binding in layer 2/3 and layer 5 of the somatosensory cortex (SSC) in adolescent and adult mice at ZT0, ZT12, and ZT12 following sleep deprivation (SD). (E–H) Normalized postsynaptic spine density in layer 2/3 and layer 5 of the SSC in adolescent and adult mice at ZT0, ZT12, and ZT12 following sleep deprivation (SD). Normalization was calculated relative to the mean value at ZT0 using the formula: $\%Difference_{(i)} = (value_{(I)} - mean_{(ZT0)}) / mean_{(ZT0)} * 100\%$. Data are represented as mean ± SEM.
